# The Atrium in Atrial Fibrillation – A Clinical Review on How to Manage Atrial Fibrotic Substrates

**DOI:** 10.3389/fcvm.2022.879984

**Published:** 2022-07-04

**Authors:** Pedro Silva Cunha, Sérgio Laranjo, Jordi Heijman, Mário Martins Oliveira

**Affiliations:** ^1^Arrhythmology, Pacing and Electrophysiology Unit, Cardiology Service, Santa Marta Hospital, Central Lisbon Hospital University Center, Lisbon, Portugal; ^2^Lisbon School of Medicine, Universidade de Lisboa, Lisbon, Portugal; ^3^Comprehensive Health Research Center, Universidade NOVA de Lisboa, Lisbon, Portugal; ^4^Department of Cardiology, Cardiovascular Research Institute Maastricht, Maastricht University, Maastricht, Netherlands

**Keywords:** fibrotic atrial myopathy, pathophysiology, catheter ablation, atrial substrate, atrial fibrillation

## Abstract

Atrial fibrillation (AF) is the most common sustained arrhythmia in the population and is associated with a significant clinical and economic burden. Rigorous assessment of the presence and degree of an atrial arrhythmic substrate is essential for determining treatment options, predicting long-term success after catheter ablation, and as a substrate critical in the pathophysiology of atrial thrombogenesis. Catheter ablation of AF has developed into an essential rhythm-control strategy. Nowadays is one of the most common cardiac ablation procedures performed worldwide, with its success inversely related to the extent of atrial structural disease. Although atrial substrate evaluation remains complex, several diagnostic resources allow for a more comprehensive assessment and quantification of the extent of left atrial structural remodeling and the presence of atrial fibrosis. In this review, we summarize the current knowledge on the pathophysiology, etiology, and electrophysiological aspects of atrial substrates promoting the development of AF. We also describe the risk factors for its development and how to diagnose its presence using imaging, electrocardiograms, and electroanatomic voltage mapping. Finally, we discuss recent data regarding fibrosis biomarkers that could help diagnose atrial fibrotic substrates.

## Introduction

Atrial fibrillation (AF) is the most common sustained arrhythmia and is associated with a substantial economic burden and significant morbidity and mortality (1, 2). AF can be asymptomatic or lead to symptoms such as palpitations, dyspnoea, and dizziness. The condition is associated with an increased risk of serious complications, including stroke (3), dementia (4), ventricular dysfunction, and death (3, 5). With a rising prevalence, it is estimated to affect nearly 17 million people in Europe by 2030, primarily driven by the aging of the population and increased survival with chronic cardiovascular diseases (3, 6–9).

In the past two decades, the knowledge of AF pathophysiology has led to significant developments in the treatment options, particularly regarding catheter ablation (10–13). Paroxysmal forms of AF are thought to primarily depend on triggers, primarily from the pulmonary veins (PV), while persistent forms involve a more significant modification of the atrial substrate (14, 15), promoting multiple re-entrant waves that maintain the arrhythmia.

Since a significant percentage of AF patients may have an indication for catheter ablation, analysis of the potential arrhythmogenic substrate is an essential part of the clinical evaluation of AF patients. Moreover, identifying the various risk factors promoting the development of a fibrotic substrate will enable a comprehensive approach to correct these factors, thus preventing the future progression of the arrhythmic substrate and increasing long-term therapeutic success.

Fibrotic atrial cardiomyopathy (FAC), a clinical entity proposed by Kottkamp (16), and one of the EHRAS atrial cardiomyopathy consensus classes (17), is a primary form of atrial pathology, characterized by extensive fibrosis as the substrate underlying atrial arrhythmias. This concept has been evolving ever since, and some authors (18) have used it more broadly to define significant atrial fibrosis due to several insults from different aetiologies. Understanding the multiple factors and mechanisms contributing to the complex development of atrial fibrosis and the management of AF based on arrhythmogenic substrates represents a challenge for interventional electrophysiology. At the same time, it may contribute to a more personalized approach, as the presence of atrial fibrosis and its characterization may guide the operator to modify the atrial substrate beyond PV isolation and estimate prognosis based on fibrosis characteristics.

Although atrial fibrosis has different clinical manifestations (like cardiac conduction disease and atrial thrombus formation), in this manuscript, we will review the role of the atrial fibrotic substrate in the context of AF. We will discuss the electrophysiology of the atria, the pathophysiology of atrial fibrillation, the molecular and genetic aspects, and the risk factors for fibrosis development. We will also review the principal elements of diagnosing the presence of atrial fibrosis using the 12-lead electrocardiogram, imaging, and electroanatomic voltage mapping and will discuss the most clinically relevant fibrosis biomarkers.

## Pathophysiology of Atrial Fibrillation in the Fibrotic Atrial Substrate

### Conceptual Framework for Atrial Fibrillation Pathophysiology

**FIGURE 1:**
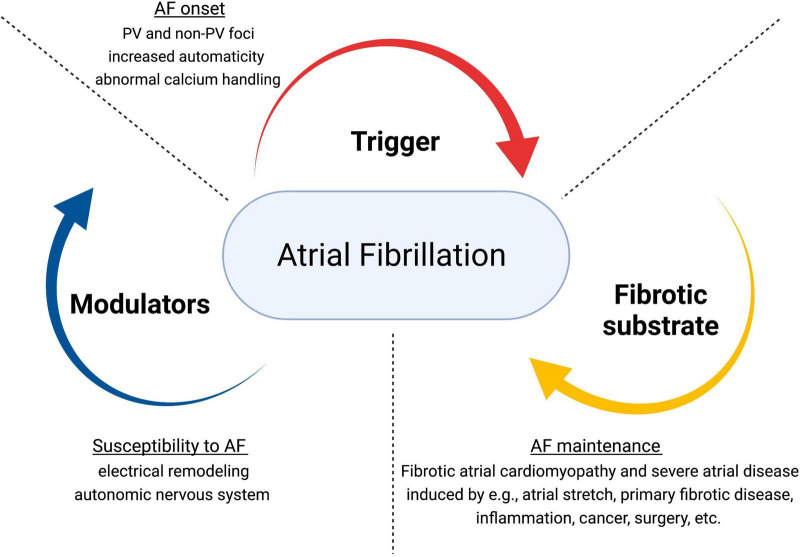
Pathophysiological dynamics in atrial fibrillation. Adapted from Kottkamp and Schreiber (24). JACC Clin Electrophysiol. AF, atrial fibrillation; PV, pulmonary veins.

Atrial fibrillation has a multi-factorial nature and complex pathogenesis. Underlying mechanisms involve structural and electrical remodeling, autonomic nervous system dysfunction (19), and calcium dysregulation (20–23). The pathophysiological triangle for AF comprises triggers (for the arrhythmia initiation), a structural (typically fibrotic) substrate (for the maintenance of AF), and different modulators (that promote the propensity to AF through multiple potential mechanisms) (9, 16, 24, 25) (Figure 1). Re-entry is considered the primary mechanism for AF maintenance. Generally, it requires a vulnerable substrate characterized by slow conduction and short effective refractory periods, combined with a trigger to initiate the unidirectional block.

Cardiac structural remodeling is characterized by atrial enlargement, a vital determinant of the persistence of AF-maintaining re-entry, and tissue fibrosis, characterized by the excessive accumulation of collagenous material in the extracellular space (12, 20, 26). Fibrotic atrial cardiomyopathy is a progressive disease with heterogeneous expressions, from mild to severe, and wide clinical variations, from asymptomatic to multiple arrhythmic manifestations (12, 16, 25, 27). Fibrosis is promoted by various risk factors (discussed below). It is involved in nearly all types of heart disease, including different ischemic and non-ischemic aetiologies (28). In many patients, AF can be understood as a manifestation of pre-existing atrial fibrosis, integrated into a gradual remodeling process (20, 27), albeit with a highly variable rate of progression determined by the dynamics of the fibrosis-promoting risk factors (29). In addition, AF itself promotes atrial fibrosis, which will contribute to AF progression and the development of therapeutic resistance in patients with long-standing arrhythmia (20, 30). Atrial fibrosis can interfere directly with impulse propagation by forming barriers to electrical conduction and separating the well-connected syncytium (31, 32). The increase in the extracellular matrix will disturb the continuity of the fibers bundle, causing local conduction disturbances (33). Additionally, direct electrical fibroblast-cardiomyocyte interactions may cause changes in cardiomyocyte electrophysiology (20, 34). Cardiac fibroblasts express multiple ion channels (35). Even though fibroblasts do not generate action potentials, they may influence cardiac electrophysiology by electrical coupling via gap junctions with cardiomyocytes (36). Finally, perivascular fibrosis around intracoronary vessels may impair oxygen and nutrient availability, promoting myocyte ischemia (37).

### Prevalence and Mechanisms of Atrial Fibrosis

**FIGURE 2:**
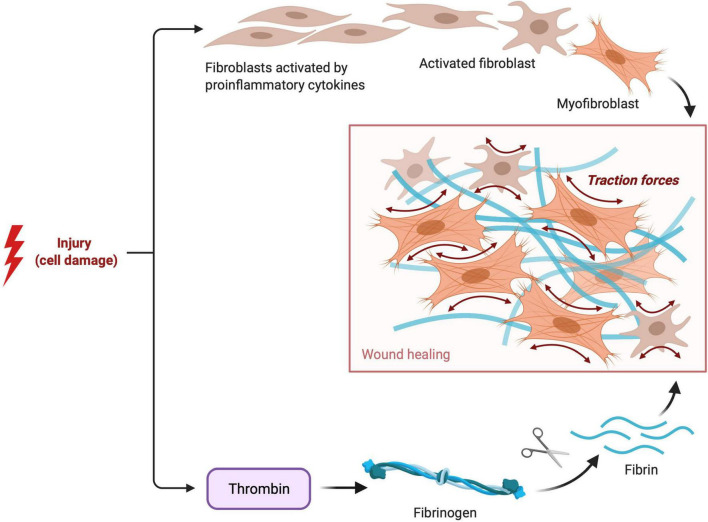
Fibroblast and Fibrin Activity in tissue healing. Visual representation of the pathophysiological process of reparative fibrosis after an injury to the cell.

Cardiac fibrosis is pathological extracellular matrix (ECM) remodeling resulting in abnormal matrix composition (38). The cardiac ECM serves as a mechanical scaffold and is involved in the transmission of contractile force (39). The ECM consists of several proteins (40) like type I collagen (the most abundant protein), type III collagen, and a wide range of glycoproteins, glycosaminoglycans, and proteoglycans, and is a reservoir of stored latent growth factors and proteases, that can be rapidly activated following injury (41). Tissue remodeling results from an imbalance in the equilibrium of the normal synthesis process and degradation of ECM components (42). Extracellular matrix deposition is a physiologic and protective process essential for wound healing (Figure 2), but excessive or prolonged deposition can impair tissue function (43).

In the normal heart, thin layers of perimysium and endomysium surround myocardial bundles and individual myocytes, respectively. The walls of the blood vessels also contain adventitial fibroblasts that contribute to the endomysial collagen network (44). In histologic analyses, two predominant types of myocardial fibrosis can be identified: interstitial fibrosis and replacement fibrosis (40). In a typical example of necrosis - myocardial infarction - necrotic cardiomyocytes are replaced by collagen-based scar, causing ‘replacement fibrosis.’ ‘Interstitial fibrosis’ (also called “reactive”) (45) describes the expansion of the endomysial and perimysial space caused by the net accumulation of ECM proteins in the absence of significant cardiomyocyte loss. The term ‘perivascular fibrosis’ is used to describe the expansion of the microvascular adventitia (46).

In the heart, ECM deposition is primarily mediated by the activation of fibroblasts in response to injury, transforming them into ECM-secreting myofibroblasts (47). Fibroblast-mediated fibrosis can affect every tissue and is a frequent pathological feature of chronic inflammatory diseases (48, 49). Similarly, expansion of the cardiac *interstitium* and deposition of ECM proteins are consistently noted in experimental models of heart failure (HF) and human patients with cardiomyopathic conditions, regardless of etiology (50).

Fibroblasts are the primary regulator of cardiac ECM. In response to disease stimuli, cardiac fibroblasts undergo cell state transitions to a myofibroblast phenotype (51). This transition is a dynamic state that underlies the fibrotic response (52). Most activated myofibroblasts in the infarcted and pressure-overloaded hearts derive from resident fibroblast populations (47). Myofibroblasts are fibroblast-smooth muscle cell hybrid that more effectively secretes and remodels the ECM positioned between all myocytes (53). Myofibroblasts have typically been defined by critical phenotypic features, including the *de novo* expression of markers including α-smooth muscle actin and periostin, increased production of ECM, and the ability to contract (54). Myofibroblasts are intimately associated with hypertrophic fibrotic scars in various injury models, and differentiation from fibroblast to myofibroblast is promoted by transforming growth factor-β (TGF-β), cytokines, the ECM, and other growth factors (55).

Several cytokines, chemokines, and growth factors are induced in the injured heart. In conjunction with elevated wall tension, specific signaling pathways and downstream effectors are mobilized to initiate myofibroblast differentiation (53). While the signaling mechanisms governing fibroblast to myofibroblast conversion are not fully elucidated, much has been discovered. Transforming growth factor β1 (TGFβ1) is considered a master regulator (56). The TGFβ-Smad signaling pathway has long been known to be involved in this process and is arguably one of the most potent inductive mechanisms (57). TGFβ drives fibroblast activation via the activation of phosphorylation of Smad2 and/or Smad3, which complex with Smad4, translocate to the nucleus and form a transcriptional complex that can directly bind to and transactivate essential ECM genes such as those encoding type I collagen (58). TGFβ may also work via a parallel non-canonical signaling pathway involving the activation of protein kinases such as p42/p44 MAPK (59). Several potential critical drivers of fibroblast activation post-MI include IL-1α/β, TGF-β1, collagen, fibronectin, osteopontin, thrombospondin-1, and secreted protein acidic and rich in cysteine (SPARC) as well as mechanical signals (e.g., scleraxis, TRPC, and MRTF/SRF) (60).

This is an area of intense and prolific investigation, leading to a rapid evolution of knowledge. Recent data on transcriptome maturation suggest that muscle blind-like 1 (MBNL1) is a post-transcriptional switch, controlling fibroblast state plasticity during cardiac wound healing (52). In this study, in healthy mice, cardiac fibroblast-specific overexpression of MBNL1 transitioned the fibroblast transcriptome to that of a myofibroblast and, after injury promoted myocyte remodeling and scar maturation.

Nonetheless, there are important existing knowledge gaps, the complete list of factors that involve fibroblast activation still needs to be identified, and the importance of individual factors ranked (51).

The gold standard for determining atrial structural remodeling is histology, which is challenging to apply in the clinical setting. Nevertheless, a few studies have included histological analyses, mainly in the surgical context. In these studies, hypertrophy of myocytes and areas of fibrosis, particularly in the left atrium, constitute the basis for AF in hypertensive patients (61). In addition, in valvular heart disease, severe fibrosis and hypertrophy with degenerative changes in atrial cardiomyocytes are the most prominent histologic findings in AF patients (62). Patients with long-standing persistent (‘chronic’) AF undergoing mitral valve surgery displayed abundant collagen fibers, inflammatory infiltrates, and sympathetic nerve twigs surrounding individual atrial cardiomyocytes, thus breaking up their clusters typically seen in sinus rhythm patients (63).

In a study investigating whether patients who develop postoperative AF show pre-existent alterations in right-atrial histopathology (64), the investigators analyzed samples from the right atrial appendage (immediately collected after opening the pericardium) from seventy patients undergoing elective coronary revascularization. The histologic abnormalities associated with the development of postoperative AF in 22 (31%) patients were interstitial fibrosis, vacuolization, and nuclear derangement of myocytes. In multivariate analysis, myocyte vacuolization and nuclear derangement represented independent predictors of postoperative AF.

## Molecular Aspects of Atrial Fibrogenesis

**FIGURE 3:**
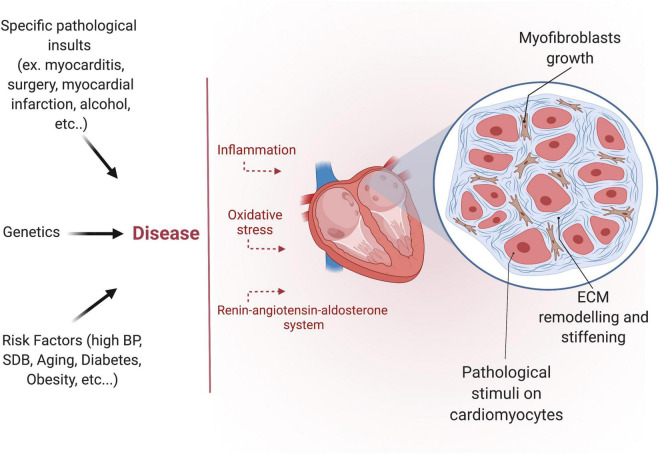
Etiology of atrial fibrosis. Different pathological insults, risk factors, and certain genetic diseases induce atrial fibrosis. Atrial fibrosis is characterized by myofibroblast growth and extracellular matrix (ECM) remodeling.

The development of fibrosis is a highly complex, multifactorial, and patient-specific process (Figure 3). Despite the growing interest in the subject over the past few years, the precise molecular mechanisms and signaling pathways involved in developing the human AF substrate are not entirely understood. Nevertheless, three interrelated signaling pathways appear to play a central role: the renin-angiotensin system (RAAS), the transforming growth factor-β1 (TGF-β1), and the oxidative stress pathways (65–67).

The RAAS plays a crucial role in cardiac structural remodeling and the development of myocardial fibrosis in several diseases states, including cardiomyopathy (65). Activation of the RAAS induces oxidative stress, which contributes to cardiovascular inflammation, fibrosis, and dysfunction (68). Angiotensin-converting enzyme (ACE) overexpression results in atrial fibrosis in several animal models (69–71), whereas the use of ACE inhibitors delays atrial fibrosis and reduces AF vulnerability and AF progression (65, 72, 73). In the right atrial tissue of patients undergoing open-heart surgery (74), the increase of atrial collagen deposition observed in atrial samples from AF patients undergoing open-heart surgery was also attenuated in those previously under ACE-inhibitor therapy, and the atrial micro-capillary density in these patients was similar to patients in normal sinus rhythm. In agreement, other studies have shown that ACE-inhibitor therapy is associated with a significant reduction in recurrent AF (75–77).

TGF-β1 is implicated in tissue repair and development of fibrosis, including atrial myocardial fibrosis, by enhancing collagen synthesis (18).

Inflammation has been implicated in various AF-related pathological processes, including oxidative stress, fibrosis, and thrombogenesis (78). Inflammation and oxidative stress may promote AF, as suggested by increased C-reactive protein (CRP) and evidence of oxidative injury seen during AF (79–81). AF induces substantial oxidative stress in fibrillating atrial tissue (14).

In many diseases, tissue inflammation is a significant trigger for fibrosis development (82). The inflammatory response is mediated by inflammasomes, which are intracellular multiprotein complexes that can trigger the host-defense response (83). The inflammasomes comprise a family of cytosolic pattern-recognition receptors called nucleotide-binding oligomerization domain (NOD)–like receptors (NLRs) that are involved in innate immune recognition of pathogen-associated molecular patterns as well as intracellular and extracellular damage-associated molecular patterns (84). Functionally, inflammasomes are sensors and receptors of the innate immune system that can induce inflammation in response to pathogens and molecules derived from host proteins. In response to these “cellular danger signals,” the inflammasomes activate caspase-1 and release both IL-1β and IL-18 via pores formed by the N-termini of gasdermin-D, which are cleaved by activated caspase-1 (85). Activation of the NLR family pyrin domain containing 3 (NLRP3) inflammasome is increased in patients with paroxysmal and long-standing-persistent AF (86), patients that go on to develop post-operative AF (87), and patients with risk factors for AF such as diabetes and obesity (88, 89) via both priming (increased expression of components of the NLRP3 inflammasome) and triggering (assembly of the NLRP3 complex) mechanisms. Activating NLRP3 selectively in atrial cardiomyocytes is sufficient to promote atrial structural remodeling (atrial hypertrophy), spontaneous premature atrial contractions, and inducible AF (86). The previously cited and fascinating clinical study (87) analyzed tissue from patients with postoperative AF, preexisting Ca^2+^-handling abnormalities, and activation of NLRP3-inflammasome/CaMKII signaling were evident in atrial cardiomyocytes.

Inflammasome signaling and downstream cytokine responses mediated by the inflammasome have been found to play an important role not only in wound healing but also in fibrosis.

Inflammasome activation induces the differentiation of quiescent fibroblasts to myofibroblasts (84). In addition, it is hypothesized that chronic dysregulation of the inflammasome promotes the differentiation of myofibroblasts, leading to excessive extracellular matrix accumulation and subsequent failure of the affected organ (90). The inflammasome regulates the secretion of IL-1β and IL-18 cytokines (91), and both are critical for repairing damaged tissue and play a role in fibrosis. Inflammasome-mediated activation of IL-18 in the myocardium is a crucial trigger for the cytokine cascade and macrophage infiltration in the heart, leading to adverse cardiac remodeling (92). However, what dictates the delicate balance between routine wound healing versus fibrosis is yet to be fully elucidated (90).

Several studies have linked fibrosis to perturbations in cardiac (myo)fibroblast calcium (Ca^2+^) handling and electrophysiology, providing a basis for future investigation of molecular targets for the prevention of fibrosis progression (93, 94). For example, transient receptor potential (TRP) channel remodeling has been implicated in profibrotic atrial remodeling in large animal models and human samples. TRP melastatin-related 7 (TRPM7) is a Ca^2+^-permeable channel upregulated in atrial fibroblasts from AF patients, likely in a TGF-β1-dependent manner (95). TRPM7 downregulation reduced basal AF fibroblast differentiation as well as TGF-β1 induced fibroblast differentiation in culture (95). Similarly, TRP canonical 3 (TRPC3) expression is upregulated in atria from AF patients, goats with electrically maintained AF, and dogs with tachypacing-induced HF, whereas TRPC3 knockdown decreased canine atrial fibroblast proliferation (96). Moreover, *in vivo* administration of the TRPC3 blocker pyrazole-3 suppressed AF in dogs while decreasing fibroblast proliferation and extracellular matrix gene expression (96). Various molecules have been associated with disturbances in atrial Ca^2+^ handling in AF. Patients with AF have elevated atrial endothelin-1 levels, associated with increased atrial preexcitation (97), inadequate Ca^2+^ leak, and increased intracellular overload. Additionally, mice with cardiac-specific knockout of liver kinase B1 (LKB1), a protein highly expressed in the heart and responsible for regulating myofilament response to Ca^2+^, developed early-onset atrial cardiomyopathy (98). Fibrosis progression has also been associated with atrial ion channel remodeling (36). Wiedmann et al. (99) showed that the TASK-1 [two-pore-domain potassium (K^+^) channel that contributes to the regulation of atrial action potential duration] is decreased in AF-prone transgenic mice, leading to both FAC and AF progression. For some K^+^ channels expressed in atrial fibroblasts, their profibrotic effects have been attributed to increasing the driving force for fibroblast Ca^2+^ entry, e.g., in the case of HF-related upregulation of KCNJ2, underlying the inward-rectifier K^+^ current (100). Similarly, mutations in the voltage-gated sodium channel have been associated with LA dilatation (101).

## Genetic Basis of Atrial Fibrillation

Atrial fibrillation has precise genetic determinants, including common and rare gene variants with variable penetrance (17, 102–104). Over the last decades, multiple studies have observed familial aggregation of individuals with lone AF (105). A family history of AF in a first-degree relative independently increases AF risk twofold (7), with the most substantial risks associated with young age at AF onset and multiple affected relatives (106). Genome-wide association studies have identified genetic variants associated with increased susceptibility to atrial fibrillation, with the strongest hits clustering on chromosome 4q25, close to the gene for the homeobox transcription factor PITX2 and single nucleotide polymorphisms in T-box (TBX)5 (107–110). However, in most individuals, atrial fibrillation is a complex trait reflecting the combined effects of aging, genetic predisposition, comorbidities, and environmental factors (111). Both standard and rare genetic variants increase susceptibility to AF in the presence of specific risk factors (104).

Inherited arrhythmia syndromes are commonly known as ‘channelopathies,’ highlighting that mutations in genes encoding cardiac ion channels are the predominant cause of these conditions (112). There is considerable overlap in ion channel genes responsible for causing arrhythmia syndromes between atria and ventricles, with genetic defects recognized to cause episodic arrhythmias in either chamber (113). Similarly, in a significant percentage of patients atrial dilated cardiomyopathy with the fibrotic structural substrate may represent the counterpart of idiopathic ventricular dilated cardiomyopathy, which is often of genetic origin (114). Still, very little information is about the genetic causes of specific atrial cardiomyopathy. Nevertheless, some studies have identified variants in non-ion channel genes as a cause of primary arrhythmogenic atrial cardiomyopathy in the last years. Hodgson-Zingman et al. (115) reported a genetic mutation in the atrial natriuretic peptide gene – Natriuretic Peptide Precursor A (NPPA) – in a large family with AF. They demonstrated the novel observation of the effects of this neuro-hormone on the action potential of the atrial myocardium. Subsequent work has implicated this gene mutation in inherited atrial cardiomyopathy (114). They investigated the evolving arrhythmic substrate in 5 patients with isolated arrhythmogenic atrial cardiomyopathy, caused by NPPA gene mutation, with repeated electroanatomic mapping and tomographic evaluations and reported that the evolution of the arrhythmic patterns to sinus node disease with atrial standstill was associated with giant atria with extensive areas of low voltage and atrial scarring. They concluded that the evolution of the amount and distribution of atrial scarring/fibrosis constitutes the structural substrate for the different types of atrial arrhythmias in a pure genetic model of arrhythmogenic atrial cardiomyopathy.

Peng et al. (116) identified a family with heritable atrial cardiomyopathy manifesting as progressive atrial−selective electromechanical dysfunction, tachyarrhythmias, and bradyarrhythmias requiring pacemaker implantation. Myosin light−chain 4 (MYL4), encoding the atrial−selective essential myosin light chain, was identified as a candidate gene. Genetically modified rat models knocking out the MYL4 gene or knocking in the human MYL4 p.E11K mutation showed early atrial fibrosis associated with enhanced proapoptotic and profibrotic signaling associated with atrial cardiomyopathy featuring atrial arrhythmia, atrial contractile failure, and atrial enlargement. The C allele and CC genotype of rs4968309 in MYL4 were also associated with AF onset and recurrence in patients after catheter ablation (117).

Interestingly, *in silico* and functional studies suggest that atrial fibrillation-associated genetic variants generate an arrhythmogenic atrial cardiomyopathic substrate (111). A better understanding of AF heritability will improve AF prediction models and be the next step toward more efficient personalized treatment strategies (118). Nevertheless, most patients have significant acquired risk factors predisposing to this fibrotic response, which we will describe in the next section.

## Risk Factors for the Development of Atrial Fibrosis

Numerous risk factors have been identified as contributors to fibrosis and AF’s development and dynamic progression. They include but are not limited to advanced age, HF, obesity, hypertension, sleep-disordered breathing, and diabetes (119).

### Aging

Incidence and prevalence of AF are age-dependent (7, 120), with increasing fibrosis being a characteristic of the aging heart (49, 121). Age-associated changes of the atria include global and regional reductions in atrial voltage with an increased heterogeneity, conduction slowing (with alterations of the wavefront propagation), prolongation of atrial refractoriness, fractionated electrograms, and double potentials (122, 123).

### Heart Failure

Atrial fibrillation and congestive HF are commonly encountered together, and each condition predisposes to the other. In the Framingham cohort (124), HF was significantly associated with AF risk in both sexes (OR, 4.5 for men and 5.9 for women). The prevalence of AF in patients with HF increases from <10% in those with New York Heart Association (NYHA) functional class I HF to approximately 50% in those with NYHA functional class IV HF (125). Moreover, diastolic dysfunction appears to be a potent precursor of AF, with an independent, graded relationship between the severity of diastolic dysfunction and the development of AF (126). Patients with HF who have concurrent AF have worse outcomes (127).

The HF and AF share common mechanisms, including myocardial fibrosis and dysregulation of intracellular calcium and neuroendocrine function (128). In animal models of HF induced by rapid ventricular pacing, there was a more significant atrial interstitial fibrosis than in AF induced by rapid atrial pacing (129). This study’s histological analysis displayed extensive interstitial fibrosis accompanied by cell loss, degenerative changes, and hypertrophy. The connective tissue was composed of increased numbers of fibroblasts, large amounts of collagen, ground substance, and occasionally fat cells. These changes were more extensive in LA. Subsequent work (130) revealed that apoptosis, leukocyte infiltration, and an increased cell death rate occur before arrhythmogenic atrial structural remodeling associated with experimental HF. These authors suggested that apoptosis is more likely associated with the pathophysiological mechanisms leading to the AF substrate rather than a result of AF *per se*.

Although both experimental paradigms promote AF, the atrial cellular electrophysiological substrate produced by HF is different from that seen with atrial tachycardia-induced remodeling. Similarly, HF and cAF produce distinct electrical and calcium-handling remodeling in human atrial samples, with repolarization shortening in cAF but not HF (131). By contrast, protein levels of ECM components are significantly increased in HF patients (131), suggesting a significant role of re-entry-promoting structural remodeling in AF development. Thus, HF promotes the presence of AF (by producing an altered substrate), and, in turn, the presence of AF worsens the prognosis of the patient with HF. It should be highlighted that CHF has different dynamic components with distinct time courses, which can further modulate the interaction between AF and HF (29).

### Obesity

There is a strong correlation between obesity and AF (132–134). In a meta-analysis of 16 studies (135), obesity increased the risk of developing AF by 49% in the general population. Additionally, obesity is usually accompanied by several other risk factors predisposing to developing AF (136). Epicardial adipose tissue is metabolically active (137), with its cardiometabolic risk being comparable to other visceral fat stores. Specifically, it can directly affect the atrial myocardium by releasing adipokines, which promote inflammation and fibrosis (138). Epicardial adipose tissue accumulation is closely associated with atrial and ventricular arrhythmias and electrocardiographic signs associated with arrhythmogenesis (139). Patients with AF have higher levels of epicardial adipose tissue than controls, and those with chronic AF are more likely to have a higher volume of epicardial adipose tissue than those with paroxysmal AF (140, 141). Volume or thickness of epicardial adipose tissue, measured on cardiac computed tomography (CT) and cardiac magnetic resonance (CMR), are predictors of the presence, severity, and recurrence of AF (142).

### Hypertension

There is a well-established association between hypertension and AF (143–146). Although, from an epidemiological perspective, it is still unclear whether the risk of AF rises linearly with blood pressure (BP) or whether there is a BP threshold above which the risk increases (147). In the Framingham study, hypertension added an excess risk for AF of 50% in men and 40% in women (124). In animal models of experimental hypertension, the high BP rapidly induced LA hypertrophy, fibrosis, and inflammation (14). In humans chronic systemic hypertension with left ventricular hypertrophy is accompanied by atrial remodeling characterized by slowing of global and regional conduction, increase in low voltage areas, and easier inducibility of sustained AF (148). LA enlargement and associated P-wave changes predict AF occurrence in hypertensive patients (149, 150). Hypertension is also a risk factor for arrhythmia recurrence after AF ablation, but it is unclear whether this is independent of other factors such as atrial size (17).

### Sleep-Disordered Breathing

Sleep-disordered breathing (SDB) has been linked to long-term adverse outcomes and is proposed as an additional and independent risk factor for cardiovascular diseases (151). SDB is highly prevalent among AF patients (from 21 to 74%), promotes arrhythmogenesis, and impairs treatment efficacy (152, 153). There is evidence that SDB – may – promote atrial fibrosis in animal models. Previous studies showed that SDB induces conduction slowing decreases matrix metalloproteinase-2, changes atrial connexin-43 expression and distribution, and significantly increases atrial fibrous tissue content (154, 155). In the clinical setting, the atria of SDB patients have extensive areas of low voltage and conduction abnormalities (156). A meta-analysis of observational studies concluded that SDB was associated with AF recurrence after catheter ablation (157). Patients with SDB had a 31% greater risk of AF recurrence after successful catheter ablation than patients without SDB. Importantly, in the same study, the efficacy of catheter ablation for AF was similar between patients without SDB and those with SDB undergoing continuous positive airway pressure treatment.

### Diabetes Mellitus

Diabetes mellitus (DM) is an independent risk factor for the development and progression of AF (158). Patients with DM have a 40% higher risk of developing AF than patients without DM (159). They have increased levels of angiotensin II, TGF-β signaling, adipose tissue, systemic inflammation Campo (160), larger atria, lower atrial voltage, and higher recurrence of AF after ablation (161). Evidence of widespread fibrotic deposits in the atria was also found in DM animal models (162).

### Sex

There are well-established sex differences in AF regarding its epidemiology, with a lower age-specific prevalence in women (women presenting at a later age) and its clinical presentation, with women more likely to be symptomatic (163). Globally the number of men and women with AF are similar since, on average, women live longer than men (164), and after 75 years of age, about 60% of the people with AF are women. Women with AF have an increased risk of stroke and death compared to men (165), which, besides differences in treatment, might in part be explained by the interesting observation that women with AF have a more significant atrial fibrosis burden, which may predispose them to more AF-associated complications. In a study with CMR in 939 patients (166), advancing age and female sex were associated with a higher burden of atrial fibrosis in AF patients. Women with a prior history of stroke also had more fibrosis than women and men without a history of stroke. In another study, female sex and AF persistence were independently associated with the presence of fibrosis on delayed enhancement CMR (167). The delayed enhancement was variably distributed in this population but more frequently detected in the posterior wall. Thus, females may have a higher probability of the presence of atrial fibrosis and atrial myopathy. Despite these observations, the mechanisms underlying differences between sexes are mainly unknown.

## Identifying Fibrotic Atrial Substrates by Electrocardiogram

The electrocardiogram (ECG) during normal sinus rhythm could be a tool to characterize the fibrotic substrate and predict AF risk (168). Interestingly in a study with 285,933 individuals (169), compared with the reference group (P wave duration of 100–105 ms), individuals with very short (≤89 ms; hazard ratio [HR] 1.60, 95% confidence interval [CI] 1.41–1.81), and very long P-wave duration (≥130 ms; HR 2.06, 95% CI 1.89–2.23) had an increased risk of incident AF.

The rationale behind using ECG as a prediction tool is that atrial remodeling is associated with an increased risk of AF and can be detected by a shift in the P-wave axis (170). The terminal force of the P wave during sinus rhythm in lead V1 (PTFV_1_) correlates with LA anomalies (171). A PTFV_1_ > 0.06 mm/s is associated with an increased risk for the development of AF (hazard ratio 4.02, 95% confidence interval 1.25–17.8; *P* = 0.018) (172, 173), and PTVF1 is independently related to cryptogenic, cardioembolic and ischemic strokes (174, 175). Suppose this evidence of inter-atrial conduction block is present in the absence of chamber enlargement or ischemia (especially in elderly patients with P-wave duration >140 ms). In that case, it can be a marker of short-term development of AF, an association called Bayés syndrome (176). Furthermore, PTFV_1_ ≥0.04 mm/s, along with P-wave duration ≥125 ms and P-wave dispersion ≥40 ms, are predictors of AF recurrence post-PVI (P wave duration >125 ms had 60% sensitivity, 90% specificity, positive predictive value of 72% and negative predictive value of 83.7%) (177).

Recently, a deep convolutional neural network trained on >1 million 12-lead resting ECGs predicted new-onset AF within 1 year (178). This model classified 62% of all patients who experienced an AF-related stroke within 3 years of the index ECG as being at high risk for new-onset AF. Thus, electrocardiographic analysis during sinus rhythm could be an additional tool to detect the presence of a vulnerable atrial substrate. However, the exact pathophysiological features and mechanisms detected by such approaches remain incompletely understood. A better insight into the underlying mechanisms may help improve early tailored treatment to prevent substrate progression and the occurrence of adverse outcomes. Even though the ECG is one of the oldest ancillary exams in cardiology, the widespread availability of standardized digital ECGs provides an opportunity for deep learning to make a significant clinical impact in cardiac electrophysiology, including characterization of the atrial cardiomyopathy (179).

## Characterizing Atrial Structural Remodeling by Non-Invasive Imaging

Non-invasive imaging is a powerful tool for identifying patients with atrial fibrosis. Echocardiography, CT, and CMR are useful for assessing the LA structure.

### Echocardiography

Echocardiography is the modality of choice for screening patients with cardiac pathologies, including those involving the LA (180). Given the non-uniform nature of remodeling (17), real-time 3D echocardiography (3DE) technology compared with CMR reference is more accurate than conventional 2D-based analyses, resulting in fewer patients with undetected atrial enlargement [3DE-derived LA Volume values showed higher correlation with CMR than 2DE measurements (*r* = 0.93 vs. *r* = 0.74 for maximal LAV; *r* = 0.88 vs. *r* = 0.82 for minimal LAV)] (181).

Increased LA size on echocardiography is associated with a higher recurrence rate of AF treated with ablation (182) and an increased risk of stroke in patients with non-valvular AF (183, 184). Assessment of LA function can be performed by pulsed-wave Doppler measurements. Certain features, like LA active relaxation and contraction, are altered in AF patients compared to subjects with sinus rhythm, regardless of LA size and age (185). Total atrial conduction time during sinus rhythm can be estimated as the interval from the beginning of the P-wave on the body-surface ECG to the peak A’-wave on the tissue-doppler imaging (TDI) tracing of the LA lateral wall on echocardiography. This echocardiography-derived PA-TDI duration reflects electrical and structural changes to the atria (186). PA-TDI is prolonged in AF patients, including those without overt cardiovascular disease (idiopathic AF) (187), and is associated with AF recurrence after ablation in paroxysmal AF patients (188). Two-dimensional speckle-tracking echocardiography, a method to quantify atrial deformation, has also been used as a sensitive marker to detect early functional remodeling before anatomical alterations occur (189). Reduced atrial strain, as calculated using speckle-tracking, has been correlated with reduced atrial compliance and increased fibrosis. In a study by Rivner et al. (168), it was reported that global compliance tended to be an independent determinant of the presence of low-voltage zones (odds ratio 1.347, *P* = 0.046) and is a predictor of the development of AF and AF recurrence after ablation.

### Cardiac Computed Tomography

Cardiac CT is a method with excellent spatial resolution compared to CMR (190), enabling accurate assessment of atrial volume and LA wall thickness. On the other hand, it has a low contrast-to-noise ratio, which reduces its ability to distinguish between normal myocardium and scar (191). Before catheter ablation, LA volume and LA asymmetry (asymmetry over 60% predicted AF recurrence with 74% sensitivity and 73% specificity), predict the likelihood of maintaining sinus rhythm post-AF ablation (192, 193). CT-based local wall deformations correlate better with extended low-voltage areas than other remodeling surrogates (194). The progression of the shape of the LA roof determined by CT correlates with the development of non-PV arrhythmic substrate in patients undergoing AF ablation (195, 196).

### Cardiac Magnetic Resonance

Cardiac magnetic resonance has become the gold standard in volumetric LA structure and function assessments.

Contrast-enhanced CMR with gadolinium is additionally used to detect atrial fibrosis (197, 198) and can non-invasively identify atrial scar, which has been shown to spatially correlate with low-voltage areas (199). Other studies have reported the feasibility of delayed-enhancement CMR to quantify fibrosis in the LA and show that a high degree of delayed enhancement in the LA is associated with a more complex and extensive ablation with AF termination as the endpoint (200). Spragg et al. (201) reported a sensitivity and specificity of LGE for discrimination of low-voltage areas of 0.84 and 0.68, respectively. Delayed-enhancement CMR correlates with surgical biopsy results and is strongly associated with AF recurrence after catheter ablation (202, 203).

Marrouche’s group introduced the Utah scoring system (204). This score classified patients by the extent of enhanced LA area into four groups: 1 (<5%), 2 (5–20%), 3 (20–35%), and 4 (>35%). In this study, procedural outcomes were predicted by the baseline LA scar burden. During follow-up, all patients in group 1 were free of AF, but in group 4, only 4% of patients remained AF free. Other studies also found that LA fibrosis detected by CMR is associated with appendage thrombus and spontaneous contrast (205) and is an independent risk factor for stroke in patients with AF (206, 207).

Technological developments have expanded CMR use, and atrial 4-dimensional flow CMR recently emerged as a novel non-invasive approach that characterizes Campo’s atrial flow dynamics. It allows measurement of 3D blood flow and the derivation of stasis maps, providing visualization and quantification of potentially thrombogenic stasis in the LA and left atrial appendage (208–211). Similarly, non-invasive digital atrial twins based on patient-specific CMR imaging can integrate anatomical, structural, and functional determinants of atrial electrophysiology and arrhythmogenesis (29). Proof-of-concept studies have shown the promise of this approach for guiding AF ablation, and initial randomized trials comparing simulation-guided versus standard clinical therapy are ongoing (119). With these recent advances, CMR imaging can provide comprehensive images of the heart in patients with various cardiac diseases, adding prognostic value (212).

## Identifying Atrial Fibrosis by Electroanatomic Voltage Mapping

Electroanatomic voltage mapping (EAVM) plays an essential role in diagnostic and therapeutic mapping and ablation in AF patients (17), providing information regarding local voltage abnormalities that may be used as a surrogate marker of myocardial health (213). Animal studies have demonstrated the histological correlation between low voltage areas (LVA) and atrial scars (214). However, the methodology for defining LVA has not been standardized, and a clear voltage threshold for abnormality has never been histologically validated (215). Several studies have compared the voltage maps with CMR findings, correlating the bipolar voltage with late gadolinium enhancement (203, 214, 216). In one of these studies, the overlap of late gadolinium enhancement areas with LVA (defined as <0.5 mV) had a sensitivity of 84% but a specificity of only 68% (201). One potentially confounding factor is the atrial rhythm during mapping, an important determinant of voltage (217). The voltage of bipolar signals during AF is significantly reduced compared to sinus rhythm (218). It has been suggested that the correlation between LVA and posterior LA delayed enhancement on CMR (219) is significantly improved when acquired during AF compared to sinus rhythm because the fixed-rate and wavefront characteristics present during sinus rhythm may not accurately reflect underlying functional vulnerabilities responsible for AF maintenance. This is an important area of investigation to improve the correlation between voltage mapping and atrial fibrosis. New technological developments like omnipole mapping and dynamic voltage attenuation may further enhance the detection of the abnormal atrial substrate (220).

During the last decade, technological developments of EAVM have helped identify different arrhythmia patterns and locations, generating new insights into the pathophysiological mechanisms of AF. In addition, progress in body-surface mapping and computer processing has allowed non-invasive mapping of atrial activation with increasing accuracy (221). For example, Metzner et al. (222) reported that non-invasive epicardial and endocardial electrophysiology systems produce comparable characterization of rotational sources with invasive mapping. A non-invasive evaluation of segmented images (223), used to construct personalized 3D models of the fibrotic atria with biophysically realistic atrial electrophysiology, demonstrated that AF in fibrotic substrates is perpetuated by re-entrant drivers (rotors). This and several other observations have led to the hypothesis that fibrillation mechanisms may exist along a continuous spectrum, with the specific electrophenotype determined by the degree of remodeling of the underlying myocardial substrate (224) particularly the extent of atrial fibrosis.

Finally, the presence of LVA could be considered in thromboembolic risk stratification, as the presence of LA LVA correlates with a higher incidence of previous stroke or the presence of pre-existing procedure-independent silent cerebral events on cerebral delayed enhancement MRI (225).

## Biomarkers of Atrial Fibrosis

Several biological markers reflecting atrial stress, inflammation, endothelial dysfunction, kidney dysfunction, and atherosclerosis have been associated with future AF events, further supporting the correlation between inflammation (and fibrosis) and atrial dysfunction in a population at risk for AF (226). The utility of these markers is the possible identification of the presence of atrial myopathy during incipient stages of the disease and the identification of ‘high-risk’ patients for thromboembolic complications and stroke. For example, inflammation and fibrosis biomarkers (CXCL16, FABP3, PIGF, and MMP-9) were higher in subjects with worse LA reservoir function (227) in a population at risk of AF. Furthermore, blood-derived biomarkers (such as markers of inflammation, coagulation activity, cardiovascular stress, myocardial injury, and cardiac and renal dysfunction) can contribute to refining risk assessment for stroke outcomes and mortality in the presence of AF (228), since currently used clinical scores (e.g., CHA_2_DS_2_-VASc) only provide modest discrimination of stroke risk. Recent studies of biomarkers in AF have shown that they significantly improve risk stratification (229, 230).

### Troponin

Elevated troponin levels have been associated with an increased incidence of AF (231–233). However, the optimal cut-off to determine the risk of AF is unclear. There are no AF primary prevention studies using troponin screening, so it is unclear how detectable troponin levels in the absence of AF will change clinical management (234). Troponin levels increase immediately after AF ablation. More significant elevation of troponin levels is related to favorable outcomes after ablation and more significant reversal of structural remodeling. In multivariate analysis, the TnT level was the only independent predictor for responders (odds ratio 90.1; 95% confidence interval 14.95–543.3; *P* < 0.0001) (235). The reason for this paradoxical observation may be the presence of healthy myocardium, as more troponin T would be released by radiofrequency ablation in a healthy LA than in a ‘sick’ LA (in which the myocardium had already degenerated into fibrous tissue) and postprocedural troponin levels may therefore reflect preservation of healthy LA myocardium.

### Natriuretic Peptides

Some studies have shown that natriuretic peptides are elevated in patients with paroxysmal AF compared with matched controls in sinus rhythm (236, 237). Natriuretic peptides levels fall rapidly after restoring sinus rhythm (238, 239). However, the usefulness of natriuretic peptide (NT-proBNP) levels to predict the maintenance of sinus rhythm after successful cardioversion remains controversial (240, 241). Nevertheless, the addition of NT-proBNP to the CHADS_2_ and CHA_2_DS_2_-VASc risk stratification models significantly improves discrimination performance (242). Hijazi et al. (228) reported an adjusted hazard ratio of 4.0 (95% confidence interval, 3.2 to 5.0; *P* < 0.001).

### Collagen

There are two significant biomarkers of collagen metabolism, the procollagen type-III N-terminal propeptide (PIIINP) and collagen type-I carboxy-terminal telopeptide (ICTP). PIIINP reflects collagen synthesis and degradation, whereas ICTP reflects collagen degradation only (243). In a large cardiovascular disease-free, multi-ethnic, and middle-aged sample population, PIIINP and ICTP predicted new onset of AF during a median follow-up of 10 years (244). A combination of circulating biomarkers reflecting excessive myocardial ICTP is also associated with higher AF prevalence, incidence, and recurrence after ablation (245). In the later study, the adjusted hazard ratio for AF recurrence was 3.4 (*p* = 0.008).

### ST2

Suppression of tumorigenicity 2 (ST2) is a member of the IL-1 receptor (IL-1R) family that plays a major role in immune and inflammatory responses (246). In recent years, knowledge about ST2’s role in the pathophysiology of cardiovascular diseases has expanded, with strong links to myocardial dysfunction, fibrosis, cardiovascular stress, and remodeling (247). Although ST2 concentrations do not improve risk discrimination in AF patients (231) its levels are of prognostic value in patients on anticoagulation (248). Concentrations of sST2 were also significantly associated with the risk of mortality, even after adjusting for the CHA2 DS2 -VASc score [HR: 1.007 (1.001–1.013); *P* = 0.014] (248). ST2 has been proposed as a screening tool to detect atrial fibrosis in AF patients to allow a more aggressive therapy (234). Likewise, ST2 may be an objective biomarker to predict the risk of arrhythmia recurrence after ablation, emergency admission, or HF events (249, 250). In a study by Kim et al. (251), mean ST2 was higher in AF, persistent AF, and symptomatic AF and decreased after ablation. Another study, in a population of patients scheduled for cryoballoon catheter ablation (252) analyzed the relationship between ST2 and recurrence of AF. ST2 was the only independent parameter predicting AF recurrence [sensitivity: 77.3%, specificity: 79.5%; area under the curve was 0.831 (*p* < 0.001)]. It might therefore be a useful marker for detecting patients with high-grade fibrosis who will benefit less from ablation.

### Galectin-3

Galectin-3 is a marker of myocardial fibrosis, which may be involved in AF-promoting atrial remodeling (253). Galectin-3 levels correlate with LA volume and are increased in AF patients (254). In a study that compared galectin-3 levels in patients with myocardial infarction and with or without AF (255) the patients with AF had higher levels of C-reactive protein (*p* < 0.01) and galectin-3 (*p* < 0.05) than those without AF. Patients with high galectin-3 had 4.4 times greater odds of having AF. Galectin-3 levels were lower in patients without AF (*p* < 0.01) than in those with permanent/persistent AF.

A recent meta-analysis showed that higher galectin-3 levels might be associated with an increased risk of AF recurrence in catheter ablation patients (256).

### Transforming Growth Factor-β1

Several stimuli that promote AF converge in increased expression levels of TGF-β1, which in turn provokes interstitial fibrosis (257). TGF-β1 is a profibrotic cytokine and a central growth factor involved in regulating atrial fibrosis (258). Upon binding to its receptor, TGF-β1 leads to the activation of intracellular signaling cascades, which ultimately alter the expression of genes involved in differentiation, chemotaxis, and proliferation (259).

High plasma levels of TGF-β1 have been correlated with increased LA volumes and reduced bipolar voltage on electroanatomic voltage mapping (260), but results regarding its effect on the incidence of AF have been contradictory (261–263).

Therefore, researchers have been actively looking for additional biomarkers to predict therapeutic failure or morbidity mortality in patients with AF in the last decades. The measurement of several of the above-described biomarkers, such as troponin and natriuretic peptides, has consistently been demonstrated to improve risk prediction in addition to the clinical risk stratification models. However, there are still several significant uncertainties concerning its real value in detecting underlying atrial disease, partly due to these biomarkers’ unspecific nature.

## Treatment

Determining the degree of the atrial fibrotic substrate is important for deciding the therapeutic strategy. This determination may help identify patients with the highest probability of success if treated with ablation therapy or, on the contrary, the population of patients with evidence of advanced disease and should be managed with medical treatment only.

### Medical Treatment

#### Preclinical and Investigational Pharmacological Agents That May Directly Modulate the Fibrotic Substrate

As previously stated, diffuse excessive production and deposition of ECM is the primary manifestation of fibrosis. Despite extensive research in this area, there is a lack of efficacious therapies for inhibiting or reversing cardiac fibrosis, mainly due to the complexity of the cell types and signaling pathways involved (264). As an example, a clinical study that sought to determine whether matrix metalloproteinase (MMP) inhibitor, PG-116800, reduced left ventricular (LV) remodeling after myocardial infarction (MI) failed to show this objective and improve clinical outcomes (265).

The TGFβ is one of the regulators in the heart remodeling after injury. By this, the targeting of the TGFβ signaling pathway (266) has been explored as a potential therapy to inhibit fibrosis. In this context, Inhibitors of TGFβ Receptors I and II (receptors that activate the TGFβ signaling) have been tested. These inhibitors have demonstrated that they reduce myocardial fibrosis in animal models of Chagas disease and myocardial infarction (267, 268). Despite these beneficial effects, increased mortality and inflammation were observed (269, 270), and in long-term inhibition, cardiac toxicity (271). Nonetheless, novel TGFβ receptor inhibitors in experimental studies to treat cardiac fibrosis revealed an improved pharmacokinetic profile (272) and minimal toxic effects (273).

In the clinical setting, Pirfenidone (a drug that is inhibitory of TGFβ signaling) is approved as an oral anti-fibrotic drug for the treatment of idiopathic pulmonary fibrosis (274), and its use in treating cardiac fibrosis is actively studied (275). The recently published PIROUETTE trial (276) showed that pirfenidone appeared to be beneficial at reducing myocardial fibrosis among patients with heart failure with preserved ejection fraction. This medication was associated with a modest reduction in myocardial fibrosis, as assessed by cardiac MRI, compared with placebo. Despite this observation, presently, its clinical significance is still undetermined.

Tranilast is a drug used to treat allergic disorders (277–279) and dermatological diseases (280) has been demonstrated to inhibit collagen deposition by inhibiting fibroblast proliferation (281) and limit TGF-β-induced collagen synthesis by keloid-derived fibroblasts (282). Suppression of TGF- β, and more specifically, its action against tumor cells, is a proven action of tranilast that could have practical therapeutic applications (283, 284). Laboratory studies conducted on various populations of fibroblasts showed that tranilast inhibited its proliferation (285, 286). Notwithstanding clinical and experimental evidence supporting the anti-fibrotic effects of tranilast, its prolonged use can have hepatic toxicity (287), limiting its clinical application.

After the observation that in Galectin-3 knockout mice, left ventricular hypertrophy was prevented and left ventricular function was ameliorated (288), the possible application of Galectin-3 inhibition as a therapeutic target capable of slowing the progression of cardiac fibrosis (289) has been considered. Up to date, no clinical studies have been published.

As it is thought that endothelin can play a role in the pathophysiology of fibrosis, the endothelin receptor blockade has been considered another potential therapeutic target, but clinical studies have shown disappointing results (290, 291).

#### Clinical Use of Pharmacological Agents That May Modulate the Fibrotic Substrate

The guidelines from the principal Cardiac Societies (292) recommend established HF therapies that target neurohumoral pathways and may reduce mortality in HF patients, at least in part through inhibition of progressive structural remodeling (Table 1). Some of these therapies have also been studied in the population of patients with AF, and data on the potential usefulness in modifying the substrate using this so-called ‘upstream therapy’ have been published. These pharmacologic agents are ACE inhibitors (as explained previously), AT1 receptor blockers, mineralocorticoid antagonists (293, 294), and β-adrenoceptor blockers. However, the results have not been consistent (295–297). Other therapies like statins have also been studied because these agents seem to exert antifibrotic effects, modulate metalloproteinases, and interact with endothelial nitric oxide synthase that protects atrial myocardium during ischemia. However, results have been either neutral or inconsistent (298–301).

**TABLE 1:** Potential antifibrotic mechanisms of currently used pharmacologic agents.

Pharmacologic agent	Target	Mechanism	Description
B eta- blockers	β−adrenergic−receptor	Blocking the effects of beta-agonism, therefore reducing sympathetic overactivity decreasing inflammation (302). Anti-inflammatory and pro-angiogenic properties (increase cytokine IL-10; decrease cytokine IL-1β) (303).	β−adrenergic−receptor agonists, including epinephrine and norepinephrine, could produce cardiac hypertrophy and fibrosis *in vivo* (304). Cardiac fibroblasts have adrenergic receptors, and stimulation of the β2−adrenergic receptor leads to increased proliferation of human and rodent cardiac fibroblasts (305, 306).
Mineralocorticoid receptors antagonists	mineralocorticoid receptors	Reduce the proinflammatory and profibrotic effects of aldosterone (307–309)	Aldosterone stimulates the expression of profibrotic molecules, such as transforming growth factor-β1 (TGF-β1), plasminogen activator inhibitor 1 (PAI-1), endothelin 1 (ET-1), placental growth factor (PGF), connective tissue growth factor (CTGF), osteopontin, and galectin-3 (310).
ACE inhibitors/Angiotensin receptor blockers	Prevent the hydrolysis of Angiotensin I to Angiotensin II	Angiotensin-converting enzyme (ACE) promotes inflammation in the heart, kidney, and vasculature through Angiotensin II as the effector (311, 312). Angiotensin II induces fibrosis via the stimulation of TGF-β (313).	ACEIs reduce inflammation and fibrosis through the reduction of IL-6 and TNF-α (312). ACEIs have an effect on reducing TGF-β1, TGF-β2, and Th2 cytokines (314). Induce the apoptosis of cardiac fibroblasts (315). Reduction of sST2 (316).
Statins	Pleiotropic effects, e.g., anti-inflammatory, antifibrotic, and immune-modulatory (317).	Statins may have a beneficial effect on various factors that promote fibrosis, such as endothelial dysfunction, VEGF, IL-6, and TNFα (318). They have been found to improve endothelial function, exert an anti-inflammatory effect and lower the expression of VEGF (319).	Reduce the differentiation of MRC5 fibroblasts into myofibroblasts (320). Induce fibroblast apoptosis (321). Suppress epithelial-mesenchymal transition (EMT) by attenuating TGF-β signaling (322, 323). Reduce the expression of transforming growth factor (TGF)-β1, connective tissue growth factor (CTGF), RhoA and cyclin D1 (324, 325). Inhibition of geranylgeranylated Rho protein (326).

Debatable results have also been reported for the effects of fish oils (327). These conflicting data may be related to diverse study populations, differences in AF history, and concomitant diseases, resulting in heterogeneous baseline remodeling, in combination with the limited reversibility of structural remodeling once it has been established (328).

### Ablation

Although pulmonary vein isolation (PVI) is very effective in maintaining sinus rhythm in patients with paroxysmal AF (329), it is much less so in persistent AF, with a reported 5-year AF freedom rate of 20% after a single and 45% after multiple procedures (330, 331). Progression of paroxysmal to persistent forms of AF occurs in 4–15% of patients per year, depending on risk factors (332–334). In recent years, several studies have shown that the earlier the treatment of patients with AF, the better the results regarding arrhythmia recurrence, hospitalization, and repeat procedures (329, 335–337). Also, early catheter ablation was superior to antiarrhythmic drug therapy in patients with drug-refractory paroxysmal AF in delaying progression to persistent AF (338). This suggests that early intervention can slow the substrate development and the progression to established atrial fibrosis and highlights a potential role for (non-invasive) characterization of the atrial substrate in guiding therapeutic decisions.

Considerable research has shown that patients with persistent AF have a more advanced atrial disease than those with paroxysmal AF (339), and different studies have shown that the extent of fibrosis before ablation is independently associated with the likelihood of AF recurrence (340). The higher the burden of atrial fibrosis, the lower the probability of sinus rhythm maintenance. These observations led to the assessment of various strategies for modifying the arrhythmic substrate beyond PVI. Clinical and experimental studies suggest that re-entrant drivers (i.e., rotors) might maintain persistent AF (341, 342). A clinical study to evaluate the relationship between fibrosis imaged by delayed-enhancement CMR and atrial electrograms in persistent AF reported that 90 percent of complex fractionated atrial electrogram sites occur at non-delayed-enhancement and patchy delayed-enhancement LA sites (343). A fascinating animal study (344), which analyzed the histological characteristics of Complex Fractionated Electrograms (CFAE) with atrial myocardial thickness and fibrosis sites, found the presence of a thicker wall and a more significant amount of fibrosis. The atrial myocardium was significantly thicker at CFAE sites (1757.5 ± 560.5 μm) than at non-CFAE sites (1279.5 ± 337.2 μm) (*p* = 0.036). At CFAE sites, it was filled with substantially more considerable fibrotic tissue than at non-CFAE sites (22.8 ± 6.9% versus 7.2 ± 4.7%, *p* < 0.001).

A 3D, biophysically detailed computational modeling study in patient-derived atrial models with individualized fibrosis distributions – derived from late enhancement CMR - showed that AF is inducible by programmed electrical stimulation in models with a sufficient amount of fibrosis. The induced AF is perpetuated by re-entrant drivers that persist in spatially confined regions. The latter areas constitute boundary zones, between fibrotic and non-fibrotic tissue characterized by high fibrosis density and entropy values (223).

Invasive electrical mapping data from a 64-pole basket catheter have been employed in a clinical computational mapping approach, revealing sustained electrical rotors and repetitive focal beats during human AF (345). Based on these observations, these authors have pioneered the CONFIRM study (346), in which the ablation in patients with persistent AF, guided by the computational mapping approach when compared with the conventional approach, showed higher freedom from AF (82.4% versus 44.9%; *p* < 0.001) after a single procedure. Although this focal impulse and rotor modulation (FIRM) approach initially gained some popularity, a meta-analysis that evaluated the results of PVI versus PVI + FIRM ablation demonstrated no therapeutic benefit of the additional focal impulse and rotor modulation approach over PVI alone (347).

A personalized substrate modification has been tested in the last decade, essentially with ablation targeting LA LVA or guided based on CMR-derived fibrosis patterns. Four strategies have been evaluated: Isolation of fibrotic areas (Box Isolation of the Fibrotic Area, BIFA) (348), homogenization of the LVA (349, 350), selective ablation of atrial LVA (351) and different combinations of part of these strategies (352). A meta-analysis that included studies with linear ablation or ablation of complex fractionated electrograms found disappointing results (353). In this meta-analysis in comparison with PVI alone, the addition of complex fractionated atrial electrograms (CFAE) ablation [RR 0.86; 95% confidence intervals (CI) 0.64, 1.16; *P* = 0.32] or left atrial linear ablation (LALA) at the roof and mitral isthmus (RR 0.64; 95% CI 0.37, 1.09; *P* = 0.10) offered no significant improvement in arrhythmia-free survival. However, adjunctive CFAE ablation was associated with significant increases (*P* < 0.05) in procedure and fluoroscopy times.

Another meta-analysis that analyzed specifically studies with a voltage-guided substrate modification by targeting LVA in addition to PVI found that this approach was more effective, safer, and with a lower proarrhythmic potential than conventional approaches (354). A common finding in different studies is that the absence of LVA identifies patients who respond well to a PVI−based ablation strategy (355).

Nonetheless, a recently published randomized controlled trial (VOLCANO trial, (356)) demonstrated that LVA ablation, in addition to PVI, had no beneficial impact on rhythm outcomes in patients with paroxysmal AF undergoing AF ablation. Patients with LVAs showed lower AF−recurrence−free survival rates (88%) than those without LVA (57%, *P* < 0.0001; C, 53%, *P* < 0.0001), and so, the presence of LVA strongly predicted AF recurrence (356). Similarly, recently presented (357) results of the DECAAFII trial suggest that fibrosis-guided ablation was not superior to conventional PVI in reducing atrial arrhythmia recurrence but significantly increased adverse events.

This is an area of active research, and probably soon, we will have data that will allow us to perform ablation tailored to the substrate observed in the particular patient. Based on the information from the different studies cited above, the most consistent finding is that the greater the degree of the atrial fibrotic substrate, the less probable is sinus rhythm maintenance.

## Conclusion

Catheter ablation for AF has developed as an important rhythm-control strategy and nowadays is one of the most common cardiac ablation procedures performed worldwide. A rigorous assessment of the presence of atrial fibrotic substrate is important for determining the treatment options and as a predictor of long-term success after catheter ablation.

In humans, the progression from paroxysmal AF to persistent forms is marked by structural alterations of the atrial tissue (358). Although the clinical phenotype (paroxysmal vs. persistent AF) typically determines therapeutic choices, it may not be the primary driver determining the success of catheter ablation treatment. Instead, the underlying factors, primarily the extent of atrial fibrosis, may be decisive.

Despite the complexity of atrial substrate evaluation, we currently have several diagnostic resources (imaging, ECG, biomarkers, etc.) that enable a comprehensive assessment and quantification of the extent of LA structural remodeling and the presence of fibrotic atrial substrates. Given the central role of fibrosis in AF pathophysiology and therapy, such a comprehensive understanding is expected to improve AF management and patient outcomes.

## Author Contributions

PC reviewed the current literature for the present manuscript, wrote the outline, composed the manuscript, and provided critical editing of the manuscript. SL performed background research for the manuscript and provided the necessary editing of the manuscript. MO and JH reviewed the literature and offered critical editing of the manuscript. All authors contributed to the article and approved the submitted version.

## Conflict of Interest

The authors declare that the research was conducted in the absence of any commercial or financial relationships that could be construed as a potential conflict of interest.

## Publisher’s Note

All claims expressed in this article are solely those of the authors and do not necessarily represent those of their affiliated organizations, or those of the publisher, the editors and the reviewers. Any product that may be evaluated in this article, or claim that may be made by its manufacturer, is not guaranteed or endorsed by the publisher.

## References

[1] Hindricks G Potpara T Dagres N Arbelo E Bax JJ Blomström-Lundqvist C 2020 ESC guidelines for the diagnosis and management of atrial fibrillation. *Eur Heart J.* (2021) 42:373–498. 32860505 10.1093/eurheartj/ehaa612

[2] Morillo CA Banerjee A Perel P Wood D Jouven X. Atrial fibrillation: the current epidemic. *J Geriatr Cardiol.* (2017) 14:195–203. 28592963 10.11909/j.issn.1671-5411.2017.03.011 PMC5460066

[3] Lip GYH Fauchier L Freedman SB Van Gelder I Natale A Gianni C Atrial fibrillation. *Nat Rev Dis Prim.* (2016) 2:1–26. 10.1038/nrdp.2016.16 27159789

[4] Kim D Yang PS Yu HT Kim TH Jang E Sung JH Risk of dementia in stroke-free patients diagnosed with atrial fibrillation: data from a population-based cohort. *Eur Heart J.* (2019) 40:2313–23. 10.1093/eurheartj/ehz386 31212315

[5] Chugh SS Havmoeller R Narayanan K Singh D Rienstra M Benjamin EJ Worldwide epidemiology of atrial fibrillation: a global burden of disease 2010 study. *Circulation.* (2014) 129:837–47. 10.1161/CIRCULATIONAHA.113.005119 24345399 PMC4151302

[6] Krijthe BP Kunst A Benjamin EJ Lip GYH Franco OH Hofman A Projections on the number of individuals with atrial fibrillation in the European Union, from 2000 to 2060. *Eur Heart J.* (2013) 34:2746–51. 10.1093/eurheartj/eht280 23900699 PMC3858024

[7] Andrade J Khairy P Dobrev D Nattel S. The clinical profile and pathophysiology of atrial fibrillation: relationships among clinical features, epidemiology, and mechanisms. *Circ Res.* (2014) 114:1453–68. 10.1161/CIRCRESAHA.114.303211 24763464

[8] Zoni-Berisso M Lercari F Carazza T Domenicucci S. Epidemiology of atrial fbrillation: European perspective. *Clin Epidemiol.* (2014) 6:213–20. 10.2147/CLEP.S47385 24966695 PMC4064952

[9] Miller JD Aronis KN Chrispin J Patil KD Marine JE Martin SS Obesity, exercise, obstructive sleep Apnea, and modifiable atherosclerotic cardiovascular disease risk factors in atrial fibrillation. *J Am Coll Cardiol.* (2015) 66:2899–906. 10.1016/j.jacc.2015.10.047 26718677

[10] Haïssaguerre M Marcus FI Fischer B Clémenty J. Radiofrequency catheter ablation in unusual mechanisms of atrial fibrillation: report of three cases. *J Cardiovasc Electrophysiol.* (1994) 5:743–51. 10.1111/j.1540-8167.1994.tb01197.x 7827713

[11] Nademanee K McKenzie J Kosar E Schwab M Sunsaneewitayakul B Vasavakul T A new approach for catheter ablation of atrial fibrillation: mapping of the electrophysiologic substrate. *J Am Coll Cardiol.* (2004) 43:2044–53. 10.1016/j.jacc.2003.12.054 15172410

[12] Kottkamp H. Human atrial fibrillation substrate: towards a specific fibrotic atrial cardiomyopathy. *Eur Heart J.* (2013) 34:2731–8. 10.1093/eurheartj/eht194 23761394

[13] Katritsis DG Gersh BJ John Camm A. Anticoagulation in atrial fibrillation - current concepts. *Arrhythm Electrophysiol Rev.* (2015) 4:100–7. 10.15420/aer.2015.04.02.100 26835109 PMC4711523

[14] Schotten U Verheule S Kirchhof P Goette A. Pathophysiological mechanisms of atrial fibrillation: a translational appraisal. *Physiol Rev.* (2011) 91:265–325. 10.1152/physrev.00031.2009 21248168

[15] Lau DH Linz D Schotten U Mahajan R Sanders P Kalman JM. Pathophysiology of paroxysmal and persistent atrial fibrillation: rotors, foci and fibrosis. *Hear Lung Circ.* (2017) 26:887–93. 10.1016/j.hlc.2017.05.119 28610723

[16] Kottkamp H. Fibrotic atrial cardiomyopathy: a specific disease/syndrome supplying substrates for atrial fibrillation, atrial tachycardia, sinus node disease, av node disease, and thromboembolic complications. *J Cardiovasc Electrophysiol.* (2012) 23:797–9. 10.1111/j.1540-8167.2012.02341.x 22554187

[17] Goette A Kalman JMM Aguinaga L Akar J Cabrera JAA Chen SAA EHRA/HRS/APHRS/SOLAECE expert consensus on atrial cardiomyopathies: definition, characterization, and clinical implication. *Europace.* (2016) 18:1455–90. 10.1093/europace/euw161 27402624 PMC6392440

[18] Hirsh BJ Copeland-Halperin RS Halperin JL. Fibrotic atrial cardiomyopathy, atrial fibrillation, and thromboembolism: mechanistic links and clinical inferences. *J Am Coll Cardiol.* (2015) 65:2239–51. 10.1016/j.jacc.2015.03.557 25998669

[19] Oliveira M Da Silva MN Geraldes V Xavier R Laranjo S Silva V Acute vagal modulation of electrophysiology of the atrial and pulmonary veins increases vulnerability to atrial fibrillation. *Exp Physiol.* (2011) 96:125–33. 10.1113/expphysiol.2010.053280 20952490

[20] Nattel S Harada M. Atrial remodeling and atrial fibrillation: recent advances and translational perspectives. *J Am Coll Cardiol.* (2014) 63:2335–45. 10.1016/j.jacc.2014.02.555 24613319

[21] Xi Y Cheng J. Dysfunction of the autonomic nervous system in atrial fibrillation. *J Thorac Dis.* (2015) 7:193–8. 10.3978/j.issn.2072-1439.2015.01.12 25713736 PMC4321068

[22] Denham NCC Pearman CMM Caldwell JLL Madders GWP Eisner DAA Trafford AWW Calcium in the pathophysiology of atrial fibrillation and heart failure. *Front Physiol.* (2018) 9:1380. 10.3389/fphys.2018.01380 30337881 PMC6180171

[23] Nattel S Heijman J Zhou L Dobrev D. Molecular basis of atrial fibrillation pathophysiology and therapy, a translational perspective. *Circ Res.* (2020) 127:51–72. 10.1161/CIRCRESAHA.120.316363 32717172 PMC7398486

[24] Kottkamp H Schreiber D. The substrate in “early persistent” atrial fibrillation arrhythmia induced, risk factor induced, or from a specific fibrotic atrial cardiomyopathy? *JACC Clin Electrophysiol.* (2016) 2:140–2. 10.1016/j.jacep.2016.02.010 29766862

[25] Kottkamp H Schreiber D Moser F Rieger A. Therapeutic approaches to atrial fibrillation ablation targeting atrial fibrosis. *JACC Clin Electrophysiol*. (2017) 3:643–53. 10.1016/j.jacep.2017.05.009 29759532

[26] Sagnard A Hammache N Sellal J-M Guenancia C. New perspective in atrial fibrillation. *J Clin Med.* (2020) 9:3713. 10.3390/jcm9113713 33228053 PMC7699334

[27] Kottkamp H Schreiber D Moser F Rieger A. Therapeutic approaches to atrial fibrillation ablation targeting atrial fibrosis. *JACC Clin Electrophysiol.* (2017) 3:643–53. 10.1016/j.jacep.2017.05.009 29759532

[28] Liu YR Ye WL Zeng XM Ren WH Zhang YQ Mei YA. K+ channels and the cAMP-PKA pathway modulate TGF-beta1-induced migration of rat vascular myofibroblasts. *J Cell Physiol.* (2008) 216:835–43. 10.1002/jcp.21464 18551429

[29] Heijman J Linz D Schotten U. Dynamics of atrial fibrillation mechanisms and comorbidities. *Annu Rev Physiol.* (2021) 83:83–106. 10.1146/annurev-physiol-031720-085307 33064962

[30] Verheule S Tuyls E Gharaviri A Hulsmans S Van Hunnik A Kuiper M Loss of continuity in the thin epicardial layer because of endomysial fibrosis increases the complexity of atrial fibrillatory conduction. *Circ Arrhythm Electrophysiol.* (2013) 6:202–11. 10.1161/CIRCEP.112.975144 23390124

[31] Li CY Zhang JR Hu WN Li SN. Atrial fibrosis underlying atrial fibrillation (review). *Int J Mol Med.* (2021) 47:1–12. 10.3892/ijmm.2020.4842 33448312 PMC7834953

[32] de Jong S van Veen TA van Rijen HV de Bakker JM. Fibrosis and cardiac arrhythmias. *J Cardiovasc Pharmacol.* (2011) 57:630–8. 21150449 10.1097/FJC.0b013e318207a35f

[33] Burstein B Comtois P Michael G Nishida K Villeneuve L Yeh YH Changes in connexin expression and the atrial fibrillation substrate in congestive heart failure. *Circ Res.* (2009) 105:1213–22. 10.1161/CIRCRESAHA.108.183400 19875729

[34] Yue L Xie J Nattel S. Molecular determinants of cardiac fibroblast electrical function and therapeutic implications for atrial fibrillation. *Cardiovasc Res.* (2011) 89:744–53. 10.1093/cvr/cvq329 20962103 PMC3039247

[35] Li GR Sun HY Chen JB Zhou Y Tse HF Lau CP. Characterization of multiple ion channels in cultured human cardiac fibroblasts. *PLoS One.* (2009) 4:e7307. 10.1371/journal.pone.0007307 19806193 PMC2751830

[36] Jakob D Klesen A Darkow E Kari FA Beyersdorf F Kohl P Heterogeneity and remodeling of ion currents in cultured right atrial fibroblasts from patients with sinus rhythm or atrial fibrillation. *Front Physiol.* (2021) 12:663. 10.3389/fphys.2021.673891 34149453 PMC8209389

[37] Souders CA Bowers SL Baudino TA. Cardiac fibroblast: the renaissance cell. *Circ Res.* (2009) 105:1164–76. 10.1161/CIRCRESAHA.109.209809 19959782 PMC3345531

[38] Cox TR Erler JT. Remodeling and homeostasis of the extracellular matrix: implications for fibrotic diseases and cancer. *Dis Model Mech*. (2011) 4:165–78. 10.1242/dmm.004077 21324931 PMC3046088

[39] Frangogiannis NG. Cardiac fibrosis: cell biological mechanisms, molecular pathways and therapeutic opportunities. *Mol Aspects Med.* (2019) 65:70–99. 10.1016/j.mam.2018.07.001 30056242

[40] Weber KT. Cardiac interstitium in health and disease: the fibrillar collagen network. *J Am Coll Cardiol.* (1989) 13:1637–52. 10.1016/0735-1097(89)90360-4 2656824

[41] Jugdutt BI. Ventricular remodeling after infarction and the extracellular collagen matrix: when is enough enough? *Circulation.* (2003) 108:1395–403. 10.1161/01.CIR.0000085658.98621.49 12975244

[42] Robert S Gicquel T Victoni T Valença S Barreto E Bailly-Maître B Involvement of matrix metalloproteinases (MMPs) and inflammasome pathway in molecular mechanisms of fibrosis. *Biosci Rep.* (2016) 36:e00360. 10.1042/BSR20160107 27247426 PMC4945993

[43] Berk BC Fujiwara K Lehoux S. ECM remodeling in hypertensive heart disease. *J Clin Invest.* (2007) 117:568–75. 10.1172/JCI31044 17332884 PMC1804378

[44] Biernacka A Frangogiannis NG. Aging and cardiac fibrosis. *Aging Dis.* (2011) 2:158–73. 21837283 PMC3153299

[45] Verheule S Schotten U. Electrophysiological consequences of cardiac fibrosis. *Cells.* (2021) 10:3220. 10.3390/cells10113220 34831442 PMC8625398

[46] Frangogiannis NG. Cardiac fibrosis. *Cardiovasc Res.* (2021) 117:1450–88. 10.1093/cvr/cvaa324 33135058 PMC8152700

[47] Shinde AV Frangogiannis NG. Mechanisms of fibroblast activation in the remodeling myocardium. *Curr Pathobiol Rep.* (2017) 5:145–52. 10.1007/s40139-017-0132-z 29057165 PMC5646705

[48] D’Urso M Kurniawan NA. Mechanical and physical regulation of fibroblast–myofibroblast transition: from cellular mechanoresponse to tissue pathology. *Front Bioeng Biotechnol.* (2020) 8:1459. 10.3389/fbioe.2020.609653 33425874 PMC7793682

[49] Dzeshka MS Lip GY Snezhitskiy V Shantsila E. Cardiac fibrosis in patients with atrial fibrillation: mechanisms and clinical implications. *J Am Coll Cardiol*. (2015) 66:943–59. 10.1016/j.jacc.2015.06.1313 26293766

[50] Calderone A Bel-Hadj S Drapeau J El-Helou V Gosselin H Clement R Scar myofibroblasts of the infarcted rat heart express natriuretic peptides. *J Cell Physiol.* (2006) 207:165–73. 10.1002/jcp.20548 16270351

[51] Bretherton R Bugg D Olszewski E Davis J. Regulators of cardiac fibroblast cell state. *Matrix Biol.* (2020) 9:117–35. 10.1016/j.matbio.2020.04.002 32416242 PMC7789291

[52] Bugg D Bailey LRJ Bretherton RC Beach KE Reichardt IM Robeson KZ MBNL1 drives dynamic transitions between fibroblasts and myofibroblasts in cardiac wound healing. *Cell Stem Cell.* (2022) 29:419–33.e10. 10.1016/j.stem.2022.01.012 35176223 PMC8929295

[53] Davis J Molkentin JD. Myofibroblasts: trust your heart and let fate decide. *J Mol Cell Cardiol.* (2014) 70:9–18. 10.1016/j.yjmcc.2013.10.019 24189039 PMC3995855

[54] Bagchi RA Roche P Aroutiounova N Espira L Abrenica B Schweitzer R The transcription factor scleraxis is a critical regulator of cardiac fibroblast phenotype. *BMC Biol.* (2016) 14:21. 10.1186/s12915-016-0243-8 26988708 PMC4794909

[55] Walker GA Masters KS Shah DN Anseth KS Leinwand LA. Valvular myofibroblast activation by transforming growth factor-beta: implications for pathological extracellular matrix remodeling in heart valve disease. *Circ Res.* (2004) 95:253–60. 10.1161/01.RES.0000136520.07995.aa 15217906

[56] Meng XM Nikolic-Paterson DJ Lan HY. TGF-β: the master regulator of fibrosis. *Nat Rev Nephrol.* (2016) 12:325–38. 10.1038/nrneph.2016.48 27108839

[57] Czubryt MP. Cardiac fibroblast to myofibroblast phenotype conversion-an unexploited therapeutic target. *J Cardiovasc Dev Dis.* (2019) 6:28. 10.3390/jcdd6030028 31426390 PMC6787657

[58] Roche PL Filomeno KL Bagchi RA Czubryt MP. Intracellular signaling of cardiac fibroblasts. *Compr Physiol.* (2015) 5:721–60. 25880511 10.1002/cphy.c140044

[59] Zeglinski MR Roche P Hnatowich M Jassal DS Wigle JT Czubryt MP TGFbeta1 regulates scleraxis expression in primary cardiac myofibroblasts by a Smad-independent mechanism. *Am J Physiol Heart Circ Physiol.* (2016) 310:H239–49. 10.1152/ajpheart.00584.2015 26566727

[60] Ma Y Iyer RP Jung M Czubryt MP Lindsey ML. Cardiac fibroblast activation post-myocardial infarction: current knowledge gaps. *Trends Pharmacol Sci.* (2017) 38:448–58. 10.1016/j.tips.2017.03.001 28365093 PMC5437868

[61] Chimenti C Russo MA Carpi A Frustaci A. Histological substrate of human atrial fibrillation. *Biomed Pharmacother.* (2010) 64:177–83. 10.1016/j.biopha.2009.09.017 20006465

[62] Corradi D Callegari S Benussi S Maestri R Pastori P Nascimbene S Myocyte changes and their left atrial distribution in patients with chronic atrial fibrillation related to mitral valve disease. *Hum Pathol.* (2005) 36:1080–9. 10.1016/j.humpath.2005.07.018 16226107

[63] Nguyen BL Fishbein MC Chen LS Chen PS Masroor S. Histopathological substrate for chronic atrial fibrillation in humans. *Heart Rhythm.* (2009) 6:454–60. 10.1016/j.hrthm.2009.01.010 19324302 PMC2662134

[64] Mariscalco G Engström K Ferrarese S Cozzi G Bruno V Sessa F Relationship between atrial histopathology and atrial fibrillation after coronary bypass surgery. *J Thorac Cardiovasc Surg.* (2006) 131:1364–72. 10.1016/j.jtcvs.2006.01.040 16733171

[65] Everett TH IVth Olgin JEE. Atrial fibrosis and the mechanisms of atrial fibrillation. *Heart Rhythm.* (2007) 4:22–4. 10.1016/j.hrthm.2006.12.040 17336879 PMC1850572

[66] Chen Y Surinkaew S Naud P Qi XY Gillis MA Shi YF JAK-STAT signalling and the atrial fibrillation promoting fibrotic substrate. *Cardiovasc Res.* (2017) 113:310–20. 10.1093/cvr/cvx004 28158495 PMC5852635

[67] Allemani C Matsuda T Di Carlo V Harewood R Matz M Nikšić M Global surveillance of trends in cancer survival 2000–14 (CONCORD-3): analysis of individual records for 37 513 025 patients diagnosed with one of 18 cancers from 322 population-based registries in 71 countries. *Lancet.* (2018) 391:1023–75. 10.1016/S0140-6736(17)33326-3 29395269 PMC5879496

[68] Jia G Aroor AR Hill MA Sowers JR. Role of renin-angiotensin-aldosterone system activation in promoting cardiovascular fibrosis and stiffness. *Hypertension.* (2018) 72:1–12. 10.1161/HYPERTENSIONAHA.118.11065 29987104 PMC6202147

[69] Shi Y Li D Tardif JC Nattel S. Enalapril effects on atrial remodeling and atrial fibrillation in experimental congestive heart failure. *Cardiovasc Res.* (2002) 54:456–61. 10.1016/s0008-6363(02)00243-2 12062350

[70] Xiao HD Fuchs S Campbell DJ Lewis W Dudley SC Jr Kasi VS Mice with cardiac-restricted angiotensin-converting enzyme (ACE) have atrial enlargement, cardiac arrhythmia, and sudden death. *Am J Pathol.* (2004) 165:1019–32. 10.1016/S0002-9440(10)63363-9 15331425 PMC1618615

[71] Li D Shinagawa K Pang L Leung TK Cardin S Wang Z Effects of angiotensin-converting enzyme inhibition on the development of the atrial fibrillation substrate in dogs with ventricular tachypacing-induced congestive heart failure. *Circulation.* (2001) 104:2608–14. 10.1161/hc4601.099402 11714658

[72] Sakabe M Fujiki A Nishida K Sugao M Nagasawa H Tsuneda T Enalapril prevents perpetuation of atrial fibrillation by suppressing atrial fibrosis and over-expression of connexin43 in a canine model of atrial pacing-induced left ventricular dysfunction. *J Cardiovasc Pharmacol.* (2004) 43:851–9. 10.1097/00005344-200406000-00015 15167279

[73] Li Y Li W Yang B Han W Dong D Xue J Effects of cilazapril on atrial electrical, structural and functional remodeling in atrial fibrillation dogs. *J Electrocardiol.* (2007) 40:100.e1-6. 10.1016/j.jelectrocard.2006.04.001 17067622

[74] Boldt A Scholl A Garbade J Resetar ME Mohr FW Gummert JF ACE-inhibitor treatment attenuates atrial structural remodeling in patients with lone chronic atrial fibrillation. *Basic Res Cardiol.* (2006) 101:261–7. 10.1007/s00395-005-0571-2 16382287

[75] Healey JS Baranchuk A Crystal E Morillo CA Garfinkle M Yusuf S Prevention of atrial fibrillation with angiotensin-converting enzyme inhibitors and angiotensin receptor blockers: a meta-analysis. *J Am Coll Cardiol.* (2005) 45:1832–9. 15936615 10.1016/j.jacc.2004.11.070

[76] Bhuriya R Singh M Sethi A Molnar J Bahekar A Singh PP Prevention of recurrent atrial fibrillation with angiotensin-converting enzyme inhibitors or angiotensin receptor blockers: a systematic review and meta-analysis of randomized trials. *J Cardiovasc Pharmacol Ther.* (2011) 16:178–84. 10.1177/1074248410389045 21285399

[77] Lin TT Yang YH Liao MT Tsai CT Hwang JJ Chiang FT Primary prevention of atrial fibrillation with angiotensin-converting enzyme inhibitors and angiotensin receptor blockers in patients with end-stage renal disease undergoing dialysis. *Kidney Int.* (2015) 88:378–85. 10.1038/ki.2015.96 25807037

[78] Harada M Van Wagoner DRR Nattel S. Role of Inflammation in atrial fibrillation pathophysiology and management. *Circ J.* (2015) 79:495–502. 10.1253/circj.CJ-15-0138 25746525 PMC4457364

[79] Chung MK Martin DO Sprecher D Wazni O Kanderian A Carnes CA C-reactive protein elevation in patients with atrial arrhythmias: inflammatory mechanisms and persistence of atrial fibrillation. *Circulation.* (2001) 104:2886–91. 10.1161/hc4901.101760 11739301

[80] Aviles RJ Martin DO Apperson-Hansen C Houghtaling PL Rautaharju P Kronmal RA Inflammation as a risk factor for atrial fibrillation. *Circulation.* (2003) 108:3006–10. 10.1161/01.CIR.0000103131.70301.4F 14623805

[81] Tascanov MB Tanriverdi Z Gungoren F Besli F Erkus ME Altiparmak IH Relationships between paroxysmal atrial fibrillation, total oxidant status, and DNA damage. *Rev Port Cardiol.* (2021) 40:5–10. 10.1016/j.repc.2020.05.011 33461844

[82] Mack M. Inflammation and fibrosis. *Matrix Biol.* (2018) 68-69:106–21. 10.1016/j.matbio.2017.11.010 29196207

[83] Mulay SR. Multifactorial functions of the inflammasome component NLRP3 in pathogenesis of chronic kidney diseases. *Kidney Int.* (2019) 96:58–66. 10.1016/j.kint.2019.01.014 30922667

[84] Artlett CM Sassi-Gaha S Rieger JL Boesteanu AC Feghali-Bostwick CA Katsikis PD. The inflammasome activating caspase 1 mediates fibrosis and myofibroblast differentiation in systemic sclerosis. *Arthritis Rheum.* (2011) 63:3563–74. 10.1002/art.30568 21792841

[85] Szabo G Csak T. Inflammasomes in liver diseases. *J Hepatol.* (2012) 57:642–54. 10.1016/j.jhep.2012.03.035 22634126

[86] Yao C Veleva T Scott L Jr Cao S Li L Chen G Enhanced cardiomyocyte NLRP3 inflammasome signaling promotes atrial fibrillation. Circulation. 2018 Nov 13;138(20):2227-2242. *Erratum Circ.* (2019) 139:e889. 10.1161/CIRCULATIONAHA.118.035202 29802206 PMC6252285

[87] Heijman J Muna AP Veleva T Molina CE Sutanto H Tekook M Atrial myocyte NLRP3/CaMKII nexus forms a substrate for postoperative atrial fibrillation. *Circ Res.* (2020) 127:1036–55. 10.1161/CIRCRESAHA.120.316710 32762493 PMC7604886

[88] Fender AC Kleeschulte S Stolte S Leineweber K Kamler M Bode J Thrombin receptor PAR4 drives canonical NLRP3 inflammasome signaling in the heart. *Basic Res Cardiol.* (2020) 115:10. 10.1007/s00395-019-0771-9 31912235 PMC7384378

[89] Scott L Jr Fender AC Saljic A Li L Chen X Wang X NLRP3 inflammasome is a key driver of obesity-induced atrial arrhythmias. *Cardiovasc Res.* (2021) 117:1746–59. 10.1093/cvr/cvab024 33523143 PMC8208743

[90] Artlett CM. Inflammasomes in wound healing and fibrosis. *J Pathol.* (2013) 229:157–67. 10.1002/path.4116 23023641

[91] Schroder K Tschopp J. The inflammasomes. *Cell.* (2010) 140:821–32. 10.1016/j.cell.2010.01.040 20303873

[92] Xiao H Li H Wang JJ Zhang JS Shen J An XB IL-18 cleavage triggers cardiac inflammation and fibrosis upon β-adrenergic insult. *Eur Heart J.* (2018) 39:60–9. 10.1093/eurheartj/ehx261 28549109

[93] Beneke K Molina CE. Molecular basis of atrial fibrillation initiation and maintenance. *Hearts.* (2021) 2021:170–87. 10.3390/hearts2010014

[94] Heijman J Voigt N Nattel S Dobrev D. Cellular and molecular electrophysiology of atrial fibrillation initiation, maintenance, and progression. *Circ Res.* (2014) 114:1483–99. 10.1161/CIRCRESAHA.114.302226 24763466

[95] Du J Xie J Zhang Z Tsujikawa H Fusco D Silverman D TRPM7-mediated Ca2+ signals confer fibrogenesis in human atrial fibrillation. *Circ Res.* (2010) 106:992–1003. 10.1161/CIRCRESAHA.109.206771 20075334 PMC2907241

[96] Harada M Luo X Qi XY Tadevosyan A Maguy A Ordog B Transient receptor potential canonical-3 channel-dependent fibroblast regulation in atrial fibrillation. *Circulation.* (2012) 126:2051–64. 10.1161/CIRCULATIONAHA.112.121830 22992321 PMC3675169

[97] Matsubara TJ Fujiu K. Endothelin-1 and atrial cardiomyopathy. *Int Heart J.* (2019) 60:238–40. 10.1536/ihj.19-039 30890686

[98] Ozcan C Battaglia E Young R Suzuki G. LKB1 knockout mouse develops spontaneous atrial fibrillation and provides mechanistic insights into human disease process. *J Am Heart Assoc.* (2015) 4:e001733. 10.1161/JAHA.114.001733 25773299 PMC4392447

[99] Wiedmann F Kiper AK Bedoya M Ratte A Rinné S Kraft M Identification of the A293 (AVE1231) binding site in the cardiac two-pore-domain potassium channel TASK-1: a common low affinity antiarrhythmic drug binding site. *Cell Physiol Biochem.* (2019) 52:1223–35. 10.33594/000000083 31001961

[100] Qi XY Huang H Ordog B Luo X Naud P Sun Y Fibroblast inward-rectifier potassium current upregulation in profibrillatory atrial remodeling. *Circ Res.* (2015) 116:836–45. 10.1161/CIRCRESAHA.116.305326 25608527

[101] Moreau A Janin A Millat G Chevalier P. Cardiac voltage-gated sodium channel mutations associated with left atrial dysfunction and stroke in children. *Europace.* (2018) 20:1692–8. 10.1093/europace/euy041 29579189

[102] Tucker NR Ellinor PT. Emerging directions in the genetics of atrial fibrillation. *Circ Res.* (2014) 114:1469–82. 10.1161/CIRCRESAHA.114.302225 24763465 PMC4040146

[103] Kirchhof P Benussi S Kotecha D Ahlsson A Atar D Casadei B 2016 ESC guidelines for the management of atrial fibrillation developed in collaboration with EACTS. *Eur J Cardiothorac Surg.* (2016) 50:e1–88. 10.1093/ejcts/ezw313 27663299

[104] Feghaly J Zakka P London B Macrae CA Refaat MM. Genetics of atrial fibrillation. *J Am Heart Assoc.* (2018) 7:e009884. 10.1161/JAHA.118.009884 30371258 PMC6474960

[105] Ellinor PT Yoerger DM Ruskin JN MacRae CA. Familial aggregation in lone atrial fibrillation. *Hum Genet.* (2005) 118:179–84. 10.1007/s00439-005-0034-8 16133178

[106] Øyen N Ranthe MF Carstensen L Boyd HA Olesen MS Olesen SP Familial aggregation of lone atrial fibrillation in young persons. *J Am Coll Cardiol.* (2012) 60:917–21. 10.1016/j.jacc.2012.03.046 22726627

[107] Syeda F Kirchhof P Fabritz L. PITX2-dependent gene regulation in atrial fibrillation and rhythm control. *J Physiol.* (2017) 595:4019–26. 10.1113/JP273123 28217939 PMC5471504

[108] Bai J Zhu Y Lo A Gao M Lu Y Zhao J In silico assessment of class I antiarrhythmic drug effects on pitx2-induced atrial fibrillation: insights from populations of electrophysiological models of human atrial cells and tissues. *Int J Mol Sci.* (2021) 22:1265. 10.3390/ijms22031265 33514068 PMC7866025

[109] Guo DF Li RG Yuan F Shi HY Hou XM Qu XK TBX5 loss-of-function mutation contributes to atrial fibrillation and atypical Holt-Oram syndrome. *Mol Med Rep.* (2016) 13:4349–56. 10.3892/mmr.2016.5043 27035640

[110] Ma JF Yang F Mahida SN Zhao L Chen X Zhang ML TBX5 mutations contribute to early-onset atrial fibrillation in Chinese and caucasians. *Cardiovasc Res.* (2016) 109:442–50. 10.1093/cvr/cvw003 26762269 PMC4752043

[111] Fatkin D Huttner IG Johnson R. Genetics of atrial cardiomyopathy. *Curr Opin Cardiol.* (2019) 34:275–81. 10.1097/HCO.0000000000000610 30672791

[112] Marsman RF Tan HL Bezzina CR. Genetics of sudden cardiac death caused by ventricular arrhythmias. *Nat Rev Cardiol.* (2014) 11:96–111. 10.1038/nrcardio.2013.186 24322550

[113] Roberts JD Gollob MH. Impact of genetic discoveries on the classification of lone atrial fibrillation. *J Am Coll Cardiol.* (2010) 55:705–12. 10.1016/j.jacc.2009.12.005 20170805

[114] Disertori M Quintarelli S Grasso M Pilotto A Narula N Favalli V Autosomal recessive atrial dilated cardiomyopathy with standstill evolution associated with mutation of natriuretic peptide precursor A. *Circ Cardiovasc Genet.* (2013) 6:27–36. 10.1161/CIRCGENETICS.112.963520 23275345

[115] Hodgson-Zingman DM Karst ML Zingman LV Heublein DM Darbar D Herron KJ Atrial natriuretic peptide frameshift mutation in familial atrial fibrillation. *N Engl J Med.* (2008) 359:158–65. 10.1056/NEJMoa0706300 18614783 PMC2518320

[116] Peng W Li M Li H Tang K Zhuang J Zhang J Dysfunction of myosin light-chain 4 (MYL4) leads to heritable atrial cardiomyopathy with electrical, contractile, and structural components: evidence from genetically-engineered rats. *J Am Heart Assoc.* (2017) 6:e007030. 10.1161/JAHA.117.007030 29080865 PMC5721782

[117] Zhong Y Tang K Li H Zhao D Kou W Xu S Rs4968309 in myosin light chain 4 (MYL4) associated with atrial fibrillation onset and predicts clinical outcomes after catheter ablation in atrial fibrillation patients without structural heart disease. *Circ J.* (2019) 83:1994–2001. 10.1253/circj.CJ-19-0415 31406021

[118] Ragab A Sitorus G Brundel B Groot N. The genetic puzzle of familial atrial fibrillation. *Front Cardiovasc Med.* (2020) 7:14. 10.3389/fcvm.2020.00014 32118049 PMC7033574

[119] Heijman J Sutanto H Crijns HJGM Nattel S Trayanova NA. Computational models of atrial fibrillation: achievements, challenges, and perspectives for improving clinical care. *Cardiovasc Res.* (2021) 117:1682–99. 10.1093/cvr/cvab138 33890620 PMC8208751

[120] Bonhorst D Mendes M Adragaþo P De Sousa J Primo J Leiria E Prevalence of atrial fibrillation in the Portuguese population aged 40 and over: the FAMA study. *Rev Port Cardiol.* (2010) 29:331–50. 20635561

[121] Neilan TG Coelho-Filho OR Shah RV Abbasi SA Heydari B Watanabe E Myocardial extracellular volume fraction from T1 measurements in healthy volunteers and mice: relationship to aging and cardiac dimensions. *JACC Cardiovasc Imaging.* (2013) 6:672–83. 10.1016/j.jcmg.2012.09.020 23643283 PMC3683385

[122] Kojodjojo P Kanagaratnam P Markides V Davies DW Peters N. Age-related changes in human left and right atrial conduction. *J Cardiovasc Electrophysiol.* (2006) 17:120–7. 10.1111/j.1540-8167.2005.00293.x 16533247

[123] Roberts-Thomson KC Kistler PM Sanders P Morton JB Haqqani HM Stevenson I Fractionated atrial electrograms during sinus rhythm: relationship to age, voltage, and conduction velocity. *Heart Rhythm.* (2009) 6:587–91. 10.1016/j.hrthm.2009.02.023 19329365

[124] Benjamin EJ Levy D Vaziri SM D’agostino RB Belanger AJ Wolf PA. Independent risk factors for atrial fibrillation in a population-based cohort: the Framingham heart study. *JAMA.* (1994) 271:840–4. 10.1001/jama.1994.03510350050036 8114238

[125] Maisel WH Stevenson LW. Atrial fibrillation in heart failure: epidemiology, pathophysiology, and rationale for therapy. *Am J Cardiol.* (2003) 91:2D–8D. 10.1016/s0002-9149(02)03373-8 12670636

[126] Tsang TS Gersh BJ Appleton CP Tajik AJ Barnes ME Bailey KR Left ventricular diastolic dysfunction as a predictor of the first diagnosed nonvalvular atrial fibrillation in 840 elderly men and women. *J Am Coll Cardiol.* (2002) 40:1636–44. 10.1016/s0735-1097(02)02373-2 12427417

[127] Mamas MA Caldwell JC Chacko S Garratt CJ Fath-Ordoubadi F Neyses L. A meta-analysis of the prognostic significance of atrial fibrillation in chronic heart failure. *Eur J Heart Fail.* (2009) 11:676–83. 10.1093/eurjhf/hfp085 19553398

[128] Heist EK Ruskin JN. Atrial fibrillation and congestive heart failure: risk factors, mechanisms, and treatment. *Prog Cardiovasc Dis.* (2006) 48:256–69. 10.1016/j.pcad.2005.09.001 16517247

[129] Li D Fareh S Leung TK Nattel S. Promotion of atrial fibrillation by heart failure in dogs: atrial remodeling of a different sort. *Circulation.* (1999) 100:87–95. 10.1161/01.cir.100.1.87 10393686

[130] Cardin S Li D Thorin-Trescases N Leung TK Thorin E Nattel S. Evolution of the atrial fibrillation substrate in experimental congestive heart failure: angiotensin-dependent and -independent pathways. *Cardiovasc Res.* (2003) 60:315–25. 10.1016/j.cardiores.2003.08.014 14613861

[131] Molina CE Abu-Taha IH Wang Q Roselló-Díez E Kamler M Nattel S Profibrotic, electrical, and calcium-handling remodeling of the atria in heart failure patients with and without atrial fibrillation. *Front Physiol.* (2018) 9:1383. 10.3389/fphys.2018.01383 30356673 PMC6189336

[132] Wang TJ Parise H Levy D D’Agostino RB Wolf PA Vasan RS Obesity and the risk of new-onset atrial fibrillation. *J Am Med Assoc.* (2004) 292:2471–7. 10.1001/jama.292.20.2471 15562125

[133] Gami AS Hodge DO Herges RM Olson EJ Nykodym J Kara T Obstructive sleep Apnea, obesity, and the risk of incident atrial fibrillation. *J Am Coll Cardiol.* (2007) 49:565–71. 10.1016/j.jacc.2006.08.060 17276180

[134] Tedrow UB Conen D Ridker PM Cook NR Koplan BA Manson JAE The long- and short-term impact of elevated body mass index on the risk of new atrial fibrillation. The WHS (women’s health study). *J Am Coll Cardiol.* (2010) 55:2319–27. 10.1016/j.jacc.2010.02.029 20488302 PMC2880879

[135] Wanahita N Messerli FH Bangalore S Gami AS Somers VK Steinberg JS. Atrial fibrillation and obesity-results of a meta-analysis. *Am Heart J.* (2008) 155:310–5. 10.1016/j.ahj.2007.10.004 18215602

[136] Abed HS Wittert GA. Obesity and atrial fibrillation. *Obes Rev.* (2013) 14:929–38. 10.1111/obr.12056 23879190

[137] Mahabadi AA Massaro JM Rosito GA Levy D Murabito JM Wolf PA Association of pericardial fat, intrathoracic fat, and visceral abdominal fat with cardiovascular disease burden: the Framingham heart study. *Eur Heart J.* (2009) 30:850–6. 10.1093/eurheartj/ehn573 19136488 PMC3693564

[138] Hatem SN Sanders P. Epicardial adipose tissue and atrial fibrillation. *Cardiovasc Res.* (2014) 102:205–13. 10.1093/cvr/cvu045 24648445

[139] Ernault AC Meijborg VMF Coronel R. Modulation of Cardiac arrhythmogenesis by epicardial adipose tissue: JACC state-of-the-art review. *J Am Coll Cardiol.* (2021) 78:1730–45. 10.1016/j.jacc.2021.08.037 34674819

[140] Shin SY Yong HS Lim HE Na JO Choi CU Choi J II Total and interatrial epicardial adipose tissues are independently associated with left atrial remodeling in patients with atrial fibrillation. *J Cardiovasc Electrophysiol.* (2011) 22:647–55. 10.1111/j.1540-8167.2010.01993.x 21235672

[141] Zhou M Wang H Chen J Zhao L. Epicardial adipose tissue and atrial fibrillation: possible mechanisms, potential therapies, and future directions. *Pacing Clin Electrophysiol.* (2020) 43:133–45. 10.1111/pace.13825 31682014

[142] Zhou P Waresi M Zhao Y Lin HC Wu B Xiong N Increased serum interleukin-6 level as a predictive biomarker for atrial fibrillation: a systematic review and meta-analysis. *Rev Port Cardiol.* (2020) 39:723–8. 10.1016/j.repc.2020.07.009 33234354

[143] Kannel WB Abbott RD Savage DD McNamara PM. Epidemiologic features of chronic atrial fibrillation. *N Engl J Med.* (1982) 306:1018–22. 10.1056/nejm198204293061703 7062992

[144] Kannel WB Wolf PA Benjamin EJ Levy D. Prevalence, incidence, prognosis, and predisposing conditions for atrial fibrillation: population-based estimates. *Am J Cardiol.* (1998) 82:2N–9N. 10.1016/s0002-9149(98)00583-9 9809895

[145] Krahn AD Manfreda J Tate RB Mathewson FAL Cuddy TE. The natural history of atrial fibrillation: Incidence, risk factors, and prognosis in the manitoba follow-up study. *Am J Med.* (1995) 98:476–84. 10.1016/S0002-9343(99)80348-9 7733127

[146] Conen D Tedrow UB Koplan BA Glynn RJ Buring JE Albert CM. Influence of systolic and diastolic blood pressure on the risk of incident atrial ribrillation in women. *Circulation.* (2009) 119:2146–52. 10.1161/CIRCULATIONAHA.108.830042 19364977 PMC2737699

[147] Verdecchia P Angeli F Reboldi G. Hypertension and atrial fibrillation: doubts and certainties from basic and clinical studies. *Circ Res.* (2018) 122:352–68. 10.1161/CIRCRESAHA.117.311402 29348255

[148] Medi C Kalman JM Spence SJ Teh AW Lee G Bader I Atrial electrical and structural changes associated with longstanding hypertension in humans: implications for the substrate for atrial fibrillation. *J Cardiovasc Electrophysiol.* (2011) 22:1317–24. 10.1111/j.1540-8167.2011.02125.x 21736657

[149] Ciaroni S Cuenoud L Bloch A. Clinical study to investigate the predictive parameters for the onset of atrial fibrillation in patients with essential hypertension. *Am Heart J.* (2000) 139:814–9. 10.1016/S0002-8703(00)90012-7 10783214

[150] Verdecchia P Reboldi GP Gattobigio R Bentivoglio M Borgioni C Angeli F Atrial fibrillation in hypertension: predictors and outcome. *Hypertension.* (2003) 41:218–23. 10.1161/01.HYP.0000052830.02773.E4 12574085

[151] Parati G Lombardi C Hedner J Bonsignore MR Grote L Tkacova R Recommendations for the management of patients with obstructive sleep apnoea and hypertension. *Eur Respir J.* (2013) 41:523–38. 10.1183/09031936.00226711 23397300

[152] Linz D Mcevoy RD Cowie MR Somers VK Nattel S Lévy P Associations of obstructive sleep Apnea with atrial fibrillation and continuous positive airway pressure treatment a review. *JAMA Cardiol.* (2018) 3:532–40. 10.1001/jamacardio.2018.0095 29541763

[153] Strotmann J Fox H Bitter T Sauzet O Horstkotte D Oldenburg O. Characteristics of sleep-disordered breathing in patients with atrial fibrillation and preserved left ventricular ejection fraction. *Clin Res Cardiol.* (2018) 107:120–9. 10.1007/s00392-017-1163-5 28942524

[154] Iwasaki YK Kato T Xiong F Shi YF Naud P Maguy A Atrial fibrillation promotion with long-term repetitive obstructive sleep apnea in a rat model. *J Am Coll Cardiol.* (2014) 64:2013–23. 10.1016/j.jacc.2014.05.077 25440097

[155] Ramos P Rubies C Torres M Batlle M Farre R Brugada J Atrial fibrosis in a chronic murine model of obstructive sleep apnea: mechanisms and prevention by mesenchymal stem cells. *Respir Res.* (2014) 15:54. 10.1186/1465-9921-15-54 24775918 PMC4012097

[156] Dimitri H Ng M Brooks AG Kuklik P Stiles MK Lau DH Atrial remodeling in obstructive sleep apnea: implications for atrial fibrillation. *Heart Rhythm.* (2012) 9:321–7. 10.1016/j.hrthm.2011.10.017 22016075

[157] Li L Wang ZW Li J Ge X Guo LZ Wang Y Efficacy of catheter ablation of atrial fibrillation in patients with obstructive sleep apnoea with and without continuous positive airway pressure treatment: a meta-analysis of observational studies. *Europace.* (2014) 16:1309–14. 10.1093/europace/euu066 24696222

[158] Huxley RR Filion KB Konety S Alonso A. Meta-analysis of cohort and case-control studies of type 2 diabetes mellitus and risk of atrial fibrillation. *Am J Cardiol.* (2011) 108:56–62. 10.1016/j.amjcard.2011.03.004 21529739 PMC3181495

[159] Ugowe FE Jackson LR II Thomas KL. Atrial fibrillation and diabetes mellitus: can we modify stroke risk through glycemic control? *Circ Arrhythm Electrophysiol.* (2019) 12:e007351. 10.1161/CIRCEP.119.007351 30995870

[160] Bohne LJ Johnson D Rose RA Wilton SB Gillis AM. The association between diabetes mellitus and atrial fibrillation: clinical and mechanistic insights. *Front Physiol.* (2019) 10:135. 10.3389/fphys.2019.00135 30863315 PMC6399657

[161] Chao TF Suenari K Chang SL Lin YJ Lo LW Hu YF Atrial substrate properties and outcome of catheter ablation in patients with paroxysmal atrial fibrillation associated with diabetes mellitus or impaired fasting glucose. *Am J Cardiol.* (2010) 106:1615–20. 10.1016/j.amjcard.2010.07.038 21094363

[162] Kato T Yamashita T Sekiguchi A Sagara K Takamura M Takata S What are arrhythmogenic substrates in diabetic rat atria? *J Cardiovasc Electrophysiol.* (2006) 17:890–4. 10.1111/j.1540-8167.2006.00528.x 16759295

[163] Andrade J Deyell M Lee A Macle L. Sex differences in atrial fibrillation. *Can J Cardiol.* (2018) 34:429–36. 10.1016/j.cjca.2017.11.022) 29455950

[164] Feinberg WM Blackshear JL Laupacis A Kronmal R Hart RG. Prevalence, age distribution, and gender of patients with atrial fibrillation. Analysis and implications. *Arch Intern Med.* (1995) 155:469–73. 7864703

[165] Volgman AS Benjamin EJ Curtis AB Fang MC Lindley KJ Naccarelli GV Women and atrial fibrillation. *J Cardiovasc Electrophysiol.* (2021) 32:2793–807. 10.1111/jce.14838 33332669 PMC8281363

[166] Akoum N Mahnkopf C Kholmovski EG Brachmann J Marrouche NF. Age and sex differences in atrial fibrosis among patients with atrial fibrillation. *EP Europace.* (2018) 20:1086–92. 10.1093/europace/eux260 29016990

[167] Cochet H Mouries A Nivet H Sacher F Derval N Denis A Age, atrial fibrillation, and structural heart disease are the main determinants of left atrial fibrosis detected by delayed-enhanced magnetic resonance imaging in a general cardiology population. *J Cardiovasc Electrophysiol.* (2015) 26:484–92. 10.1111/jce.12651 25727248

[168] Rivner H Mitrani RD Goldberger JJ. Atrial myopathy underlying atrial fibrillation. *Arrhythm Electrophysiol Rev.* (2020) 9:61–70. 10.15420/aer.2020.13 32983526 PMC7491052

[169] Nielsen JB Kühl JT Pietersen A Graff C Lind B Struijk JJ P-wave duration and the risk of atrial fibrillation: results from the Copenhagen ECG study. *Heart Rhythm.* (2015) 12:1887–95. 10.1016/j.hrthm.2015.04.026 25916567

[170] Maheshwari A Norby FL Soliman EZ Koene R Rooney M O’Neal WT Refining prediction of atrial fibrillation risk in the general population with analysis of P-wave axis (from the atherosclerosis risk in communities study). *Am J Cardiol.* (2017) 120:1980–4. 10.1016/j.amjcard.2017.08.015 28941601 PMC9835766

[171] Hancock EW Deal BJ Mirvis DM Okin P Kligfield P Gettes LS. AHA/ACCF/HRS recommendations for the standardization and interpretation of the electrocardiogram: part V: electrocardiogram changes associated with cardiac chamber hypertrophy: a scientific statement from the American heart association electrocardiography. *Circulation.* (2009) 119:e251–61. 10.1161/CIRCULATIONAHA.108.191097 19228820

[172] Ishida K Hayashi H Miyamoto A Sugimoto Y Ito M Murakami Y P wave and the development of atrial fibrillation. *Heart Rhythm.* (2010) 7:289–94. 10.1016/j.hrthm.2009.11.012 20133209

[173] Eranti A Aro AL Kerola T Anttonen O Rissanen HA Tikkanen JT Prevalence and prognostic significance of abnormal P terminal force in lead V1 of the ECG in the general population. *Circ Arrhythm Electrophysiol.* (2014) 7:1116–21. 10.1161/CIRCEP.114.001557 25381332

[174] Kamel H Hunter M Moon YP Yaghi S Cheung K Di Tullio MR Electrocardiographic left atrial abnormality and risk of stroke: northern manhattan study. *Stroke.* (2015) 46:3208–12. 10.1161/STROKEAHA.115.009989 26396031 PMC4624510

[175] Goda T Sugiyama Y Ohara N Ikegami T Watanabe K Kobayashi J P-wave terminal force in lead V1 predicts paroxysmal atrial fibrillation in acute ischemic stroke. *J Stroke Cerebrovasc Dis.* (2017) 26:1912–5. 10.1016/j.jstrokecerebrovasdis.2017.06.031 28716584

[176] Conde D Seoane L Gysel M Mitrione S Bayés De Luna A Baranchuk A. Bayés’ syndrome: the association between interatrial block and supraventricular arrhythmias. *Expert Rev Cardiovasc Ther.* (2015) 13:541–50. 10.1586/14779072.2015.1037283 25907617

[177] Salah A Zhou S Liu Q Yan H. P wave indices to predict atrial fibrillation recurrences post pulmonary vein isolation. *Arq Bras Cardiol.* (2013) 101:519–27. 10.5935/abc.20130214 24173135 PMC4106810

[178] Raghunath S Pfeifer JM Ulloa-Cerna AE Nemani A Carbonati T Jing L Deep neural networks can predict new-onset atrial fibrillation from the 12-lead electrocardiogram and help identify those at risk of AF-related stroke. *Circulation.* (2021) 143:1287–98. 10.1161/CIRCULATIONAHA.120.047829 33588584 PMC7996054

[179] Zhou S Sapp JL AbdelWahab A Trayanova N. Deep learning applied to electrocardiogram interpretation. *Can J Cardiol.* (2021) 37:17–8. 10.1016/j.cjca.2020.03.035 32649870 PMC7815317

[180] Lang RM Badano LP Victor MA Afilalo J Armstrong A Ernande L Recommendations for cardiac chamber quantification by echocardiography in adults: an update from the American society of echocardiography and the European association of cardiovascular imaging. *J Am Soc Echocardiogr.* (2015) 28:1–39.e14. 10.1016/j.echo.2014.10.003 25559473

[181] Mor-Avi V Yodwut C Jenkins C Khl H Nesser HJ Marwick TH Real-time 3D echocardiographic quantification of left atrial volume: multicenter study for validation with CMR. *JACC Cardiovasc Imaging.* (2012) 5:769–77. 10.1016/j.jcmg.2012.05.011 22897989

[182] Njoku A Kannabhiran M Arora R Reddy P Gopinathannair R Lakkireddy D Left atrial volume predicts atrial fibrillation recurrence after radiofrequency ablation: a meta-analysis. *Europace.* (2018) 20:33–42. 10.1093/europace/eux013 28444307

[183] Providência R Trigo J Paiva L Barra S. The role of echocardiography in thromboembolic risk assessment of patients with nonvalvular atrial fibrillation. *J Am Soc Echocardiogr.* (2013) 26:801–12. 10.1016/j.echo.2013.05.010 23791115

[184] Ogata T Matsuo R Kiyuna F Hata J Ago T Tsuboi Y Left atrial size and long-term risk of recurrent stroke after acute ischemic stroke in patients with nonvalvular atrial fibrillation. *J Am Heart Assoc.* (2017) 6:e006402. 10.1161/JAHA.117.006402 28862939 PMC5586470

[185] Kojima T Kawasaki M Tanaka R Ono K Hirose T Iwama M Left atrial global and regional function in patients with paroxysmal atrial fibrillation has already been impaired before enlargement of left atrium: velocity vector imaging echocardiography study. *Eur Heart J Cardiovasc Imaging.* (2012) 13:227–34. 10.1093/ejechocard/jer281 22166594

[186] Müller P Weijs B Bemelmans NMAA Mügge A Eckardt L Crijns HJGM Echocardiography-derived total atrial conduction time (PA-TDI duration): risk stratification and guidance in atrial fibrillation management. *Clin Res Cardiol.* (2021) 110:1734–42. 10.1007/s00392-021-01917-9 34453577 PMC8563556

[187] Weijs B de Vos CB Limantoro I Cheriex EC Tieleman RG Crijns HJ. The presence of an atrial electromechanical delay in idiopathic atrial fibrillation as determined by tissue Doppler imaging. *Int J Cardiol.* (2012) 156:121–2. 10.1016/j.ijcard.2012.01.024 22305816

[188] Maenosono R Mizukami N Ichiki H Oketani N Namino F Masamoto I Total atrial conduction time as a possible predictor of atrial fibrillation recurrence after catheter ablation for paroxysmal atrial fibrillation: relationship between electrical atrial remodeling and structural atrial remodeling time courses. *J Med Ultrason.* (2001) 48:295–306. 10.1007/s10396-021-01090-6 33913054

[189] Chen Y Li Z Shen X Wang W Kang Y Qiao Z Assessment of left atrial remodeling in paroxysmal atrial fibrillation with speckle tracking echocardiography: a study with an electrophysiological mapping system. *Int J Cardiovasc Imaging.* (2019) 35:451–9. 10.1007/s10554-018-1470-6 30413910

[190] Kuchynka P Podzimkova J Masek M Lambert L Cerny V Danek B The role of magnetic resonance imaging and cardiac computed tomography in the assessment of left atrial anatomy, size, and function. *Biomed Res Int.* (2015) 2015:247865. 10.1155/2015/247865 26221583 PMC4508386

[191] De Sensi F Penela D Soto-Iglesias D Berruezo A Limbruno U. Imaging techniques for the study of fibrosis in atrial fibrillation ablation: from molecular mechanisms to therapeutical perspectives. *J Clin Med.* (2021) 10:2277. 10.3390/jcm10112277 34073969 PMC8197293

[192] Nedios S Tang M Roser M Solowjowa N Gerds-Li JH Fleck E Characteristic changes of volume and three-dimensional structure of the left atrium in different forms of atrial fibrillation: predictive value after ablative treatment. *J Interv Card Electrophysiol.* (2011) 32:87–94. 10.1007/s10840-011-9591-z 21667097

[193] Nedios S Koutalas E Sommer P Arya A Rolf S Husser D Asymmetrical left atrial remodelling in atrial fibrillation: relation with diastolic dysfunction and long-term ablation outcomes. *Europace.* (2017) 19:1463–9. 10.1093/europace/euw225 27738076

[194] Nedios S Sanatkhani S Oladosu M Seewöster T Richter S Arya A Association of low-voltage areas with the regional wall deformation and the left atrial shape in patients with atrial fibrillation: a proof of concept study. *Int J Cardiol Heart Vasc.* (2021) 33:100730. 10.1016/j.ijcha.2021.100730 33718586 PMC7933256

[195] Kurotobi T Iwakura K Inoue K Kimura R Toyoshima Y Ito N The significance of the shape of the left atrial roof as a novel index for determining the electrophysiological and structural characteristics in patients with atrial fibrillation. *Europace.* (2011) 13:803–8. 10.1093/europace/eur039 21398655

[196] Mangiafico V Saberwal B Lavalle C Raharja A Ahmed Z Papageorgiou N The role of CT in detecting AF substrate. *Trends Cardiovasc Med.* (2021) 31:457–466. 10.1016/j.tcm.2020.10.004 33068722

[197] Oakes RS Badger TJ Kholmovski EG Akoum N Burgon NS Fish EN Detection and quantification of left atrial structural remodeling with delayed-enhancement magnetic resonance imaging in patients with atrial fibrillation. *Circulation.* (2009) 119:1758–67. 10.1161/CIRCULATIONAHA.108.811877 19307477 PMC2725019

[198] Mrgulescu AD Nuñez-Garcia M Alarcón F Benito EM Enomoto N Cozzari J Reproducibility and accuracy of late gadolinium enhancement cardiac magnetic resonance measurements for the detection of left atrial fibrosis in patients undergoing atrial fibrillation ablation procedures. *Europace.* (2019) 21:724–31. 10.1093/europace/euy314 30649273

[199] Zghaib T Nazarian S. New insights into the use of cardiac magnetic resonance imaging to guide decision making in atrial fibrillation management. *Can J Cardiol.* (2018) 34:1461–70. 10.1016/j.cjca.2018.07.007 30297256

[200] Seitz J Horvilleur J Lacotte J Oh-Ici D Mouhoub Y Maltret A Correlation between AF substrate ablation difficulty and left atrial fibrosis quantified by delayed−enhancement cardiac magnetic resonance. *Pacing Clin Electrophysiol.* (2011) 34:1267–77. 10.1111/j.1540-8159.2011.03148.x 21651593

[201] Spragg D Khurram I Zimmerman SL Yarmohammadi H Barcelon B Needleman M Initial experience with magnetic resonance imaging of atrial scar and co-registration with electroanatomic voltage mapping during atrial fibrillation: success and limitations. *Heart Rhythm.* (2012) 9:2003–9. 10.1016/j.hrthm.2012.08.039 23000671

[202] Marrouche NF Wilber D Hindricks G Jais P Akoum N Marchlinski F Association of atrial tissue fibrosis identified by delayed enhancement MRI and atrial fibrillation catheter ablation: the DECAAF study. *JAMA.* (2014) 311:498–506. 10.1001/jama.2014.3 24496537

[203] McGann C Akoum N Patel A Kholmovski E Revelo P Damal K Atrial fibrillation ablation outcome is predicted by left atrial remodeling on MRI. *Circ Arrhythm Electrophysiol.* (2014) 7:23–30. 10.1161/CIRCEP.113.000689 24363354 PMC4086672

[204] Mahnkopf C Badger TJ Burgon NS Daccarett M Haslam TS Badger CT Evaluation of the left atrial substrate in patients with lone atrial fibrillation using delayed-enhanced MRI: implications for disease progression and response to catheter ablation. *Heart Rhythm.* (2010) 7:1475–81. 10.1016/j.hrthm.2010.06.030 20601148 PMC3106345

[205] Akoum N Fernandez G Wilson B Mcgann C Kholmovski E Marrouche N. Association of atrial fibrosis quantified using LGE-MRI with atrial appendage thrombus and spontaneous contrast on transesophageal echocardiography in patients with atrial fibrillation. *J Cardiovasc Electrophysiol.* (2013) 24:1104–9. 10.1111/jce.12199 23844972 PMC3818287

[206] Daccarett M Badger TJJ Akoum N Burgon NSS Mahnkopf C Vergara G Association of left atrial fibrosis detected by delayed-enhancement magnetic resonance imaging and the risk of stroke in patients with atrial fibrillation. *J Am Coll Cardiol.* (2011) 57:831–8. 10.1016/j.jacc.2010.09.049 21310320 PMC3124509

[207] Boyle PM Del Álamo JC Akoum N. Fibrosis, atrial fibrillation and stroke: clinical updates and emerging mechanistic models. *Heart.* (2021) 107:99–105. 10.1136/heartjnl-2020-317455 33097562

[208] Foll D Taeger S Bode C Jung B Markl M. Age, gender, blood pressure, and ventricular geometry influence normal 3D blood flow characteristics in the left heart. *Eur Hear J Cardiovasc Imaging.* (2013) 14:366–73. 10.1093/ehjci/jes196 23002214

[209] Lee DC Markl M Ng J Carr M Benefield B Carr JC Three-dimensional left atrial blood flow characteristics in patients with atrial fibrillation assessed by 4D flow CMR. *Eur Heart J Cardiovasc Imaging.* (2016) 17:1259–68. 10.1093/ehjci/jev304 26590397 PMC5081137

[210] Markl M Lee DC Ng J Carr M Carr J Goldberger JJ. Left atrial 4-dimensional flow magnetic resonance imaging stasis and velocity mapping in patients with atrial fibrillation. *Invest Radiol.* (2016) 51:147–54. 10.1097/RLI.0000000000000219 26488375 PMC4742429

[211] Fluckiger JU Goldberger JJ Lee DC Ng J Lee R Goyal A Left atrial flow velocity distribution and flow coherence using four-dimensional FLOW MRI: a pilot study investigating the impact of age and pre- and postintervention atrial fibrillation on atrial hemodynamics. *J Magn Reson Imaging.* (2013) 38:580–7. 10.1002/jmri.23994 23292793

[212] Lee SE Nguyen C Xie Y Deng Z Zhou Z Li D Recent advances in cardiac magnetic resonance imaging. *Korean Circ J.* (2019) 49:146–59. 10.4070/kcj.2018.0246 30468040 PMC6351278

[213] Kapa S Desjardins B Callans DJ Marchlinski FE Dixit S. Contact electroanatomic mapping derived voltage criteria for characterizing left atrial scar in patients undergoing ablation for atrial fibrillation. *J Cardiovasc Electrophysiol.* (2014) 25:1044–52. 10.1111/jce.12452 24832482

[214] Harrison JL Jensen HK Peel SA Chiribiri A Grondal AK Bloch LO Cardiac magnetic resonance and electroanatomical mapping of acute and chronic atrial ablation injury: a histological validation study. *Eur Heart J.* (2014) 35:1486–95. 10.1093/eurheartj/eht560 24419806 PMC4048535

[215] Sim I Bishop M O’Neill M Williams SE. Left atrial voltage mapping: defining and targeting the atrial fibrillation substrate. *J Interv Card Electrophysiol.* (2019) 56:213–27. 10.1007/s10840-019-00537-8 31076965 PMC6900285

[216] Malcolme-Lawes LC Juli C Karim R Bai W Quest R Lim PB Automated analysis of atrial late gadolinium enhancement imaging that correlates with endocardial voltage and clinical outcomes: a 2-center study. *Heart Rhythm.* (2013) 10:1184–91. 10.1016/j.hrthm.2013.04.030 23685170 PMC3734347

[217] Ndrepepa G Schneider MAE Karch MR Weber S Schreieck J Zrenner B Impact of atrial fibrillation on the voltage of bipolar signals acquired from the left and right atria. *Pacing Clin Electrophysiol.* (2003) 26:862–9. 10.1046/j.1460-9592.2003.t01-1-00151.x 12715847

[218] Teh AW Kistler PM Lee G Medi C Heck PM Spence SJ The relationship between complex fractionated electrograms and atrial low-voltage zones during atrial fibrillation and paced rhythm. *Europace.* (2011) 13:1709–16. 10.1093/europace/eur197 21712259

[219] Qureshi NA Kim SJ Cantwell CD Afonso VX Bai W Ali RL Voltage during atrial fibrillation is superior to voltage during sinus rhythm in localizing areas of delayed enhancement on magnetic resonance imaging: an assessment of the posterior left atrium in patients with persistent atrial fibrillation. *Heart Rhythm.* (2019) 16:1357–67. 10.1016/j.hrthm.2019.05.032 31170484 PMC6722483

[220] Sim I Bishop M O’Neill M Williams SE Left atrial voltage mapping: defining and targeting the atrial fibrillation substrate. *J Interv Card Electrophysiol.* (2019) 56:213–27. 10.1007/s10840-019-00537-8 31076965 PMC6900285

[221] Haissaguerre M Hocini M Shah AJ Derval N Sacher F Jais P Noninvasive panoramic mapping of human atrial fibrillation mechanisms: a feasibility report. *J Cardiovasc Electrophysiol.* (2013) 2013:711–7. 10.1111/jce.12075 23373588

[222] Metzner A Wissner E Tsyganov A Kalinin V Schlüter M Lemes C Noninvasive phase mapping of persistent atrial fibrillation in humans: comparison with invasive catheter mapping. *Ann Noninvasive Electrocardiol.* (2018) 23:e12527. 10.1111/anec.12527 29271538 PMC6931819

[223] Zahid S Cochet H Boyle PM Schwarz EL Whyte KN Vigmond EJ Patient-derived models link re-entrant driver localization in atrial fibrillation to fibrosis spatial pattern. *Cardiovasc Res.* (2016) 110:443–54. 10.1093/cvr/cvw073 27056895 PMC4872878

[224] Ng FS Handa BS Li X Peters NS. Toward mechanism-directed electrophenotype-based treatments for atrial fibrillation. *Front Physiol.* (2020) 11:987. 10.3389/fphys.2020.00987 33013435 PMC7493660

[225] Müller P Makimoto H Dietrich JW Fochler F Nentwich K Krug J Association of left atrial low-voltage area and thromboembolic risk in patients with atrial fibrillation. *Europace.* (2018) 20:f359–65. 10.1093/europace/eux172 29016757

[226] O’Neal WT Venkatesh S Broughton ST Griffin WF Soliman EZ. Biomarkers and the prediction of atrial fibrillation: state of the art. *Vasc Health Risk Manag.* (2016) 12:297–303. 10.2147/VHRM.S75537 27486329 PMC4957677

[227] Mimbrero Guillamon M Loncaric F Loncaric F Nunno L Nunno L Tirapu L Inflammation and fibrosis biomarkers are related to atrial dysfunction in patients at risk of atrial fibrillation. *Eur Heart J.* (2020) 41:2020. 10.1093/ehjci/ehaa946.0130

[228] Hijazi Z Oldgren J Siegbahn A Granger CBB Wallentin L. Biomarkers in atrial fibrillation: a clinical review. *Eur Heart J.* (2013) 34:1475–80. 10.1093/eurheartj/eht024 23386711

[229] Rivera-Caravaca JM Marín F Vilchez JA Gálvez J Esteve-Pastor MA Vicente V Refining stroke and bleeding prediction in atrial fibrillation by adding consecutive biomarkers to clinical risk scores. *Stroke.* (2019) 50:1372–9. 10.1161/STROKEAHA.118.024305 31084333

[230] Benz AP Hijazi Z Lindbäck J Connolly SJ Eikelboom JW Oldgren J Biomarker-based risk prediction with the ABC-AF scores in patients with atrial fibrillation not receiving oral anticoagulation. *Circulation.* (2021) 143:1863–73. 10.1161/CIRCULATIONAHA.120.053100 33849281

[231] Rienstra M Yin X Larson MG Fontes JD Magnani JW McManus DD Relation between soluble ST2, growth differentiation factor-15, and high-sensitivity troponin i and incident atrial fibrillation. *Am Heart J.* (2014) 167:109–15.e2. 10.1016/j.ahj.2013.10.003 24332149 PMC3884900

[232] Filion KB Agarwal SK Ballantyne CM Eberg M Hoogeveen RC Huxley RR High-sensitivity cardiac troponin T and the risk of incident atrial fibrillation: the atherosclerosis risk in communities (ARIC) study. *Am Heart J.* (2015) 169:31–8.e3. 10.1016/j.ahj.2014.10.005 25497245 PMC4429290

[233] Hussein AA Bartz TM Gottdiener JS Sotoodehnia N Heckbert SR Lloyd-Jones D Serial measures of cardiac troponin T levels by a highly sensitive assay and incident atrial fibrillation in a prospective cohort of ambulatory older adults. *Heart Rhythm.* (2015) 12:879–85. 10.1016/j.hrthm.2015.01.020 25602173 PMC4546831

[234] Chang KWW Hsu JCC Toomu A Fox S Maisel ASS. Clinical applications of biomarkers in atrial fibrillation. *Am J Med.* (2017) 130:1351–7. 10.1016/j.amjmed.2017.08.003 28822701

[235] Yoshida K Yui Y Kimata A Koda N Kato J Baba M Troponin elevation after radiofrequency catheter ablation of atrial fibrillation: relevance to AF substrate, procedural outcomes, and reverse structural remodeling. *Heart Rhythm.* (2014) 11:1336–42. 10.1016/j.hrthm.2014.04.015 24732367

[236] Ellinor PT Low AF Patton KK Shia MA MAcRae CA. Discordant atrial natriuretic peptide and brain natriuretic peptide levels in lone atrial fibrillation. *J Am Coll Cardiol.* (2005) 45:82–6. 10.1016/J.JACC.2004.09.045 15629379

[237] Shelton RJ Clark AL Goode K Rigby AS Cleland JGF. The diagnostic utility of N-terminal pro-B-type natriuretic peptide for the detection of major structural heart disease in patients with atrial fibrillation. *Eur Heart J.* (2006) 27:2353–61. 10.1093/eurheartj/ehl233 16952921

[238] Wozakowska-Kapłon B. Effect of sinus rhythm restoration on plasma brain natriuretic peptide in patients with atrial fibrillation. *Am J Cardiol.* (2004) 93:1555–8. 10.1016/j.amjcard.2004.03.013 15194036

[239] Yamada T Murakami Y Okada T Okamoto M Shimizu T Toyama J Plasma atrial natriuretic peptide and brain natriuretic peptide levels after radiofrequency catheter ablation of atrial fibrillation. *Am J Cardiol.* (2006) 97:1741–4. 10.1016/j.amjcard.2005.12.071 16765125

[240] Lellouche N Berthier R Mekontso-Dessap A Braconnier F Monin JL Duval AM Usefulness of plasma B-type natriuretic peptide in predicting recurrence of atrial fibrillation one year after external cardioversion. *Am J Cardiol.* (2005) 95:1380–2. 10.1016/j.amjcard.2005.01.090 15904651

[241] Freynhofer MK Jarai R Höchtl T Bruno V Vogel B Aydinkoc K Predictive value of plasma Nt-proBNP and body mass index for recurrence of atrial fibrillation after cardioversion. *Int J Cardiol.* (2011) 149:257–9. 10.1016/j.ijcard.2011.02.043 21377224

[242] Berg DD Ruff CT Jarolim P Giugliano RP Nordio F Lanz HJ Performance of the ABC scores for assessing the risk of stroke or systemic embolism and bleeding in patients with atrial fibrillation in ENGAGE AF-TIMI 48. *Circulation.* (2019) 139:760–71. 10.1161/CIRCULATIONAHA.118.038312 30586727 PMC6363338

[243] López B González A Díez J. Circulating biomarkers of collagen metabolism in cardiac diseases. *Circulation.* (2010) 121:1645–54. 10.1161/CIRCULATIONAHA.109.912774 20385961

[244] Duprez DAA Heckbert SRR Alonso A Gross MDD Ix JHH Kizer JRR Collagen biomarkers and incidence of new onset of atrial fibrillation in subjects with no overt cardiovascular disease at baseline. *Circ Arrhythm Electrophysiol.* (2018) 11:e006557. 10.1161/CIRCEP.118.006557 30354407 PMC9679990

[245] Ravassa S Ballesteros G López B Ramos P Bragard J González A Combination of circulating type I collagen-related biomarkers is associated with atrial fibrillation. *J Am Coll Cardiol.* (2019) 73:1398–410. 10.1016/j.jacc.2018.12.074 30922470

[246] Kalstad AA Myhre PL Laake K Opstad TB Tveit A Solheim S Biomarkers of ageing and cardiac remodeling are associated with atrial fibrillation. *Scand Cardiovasc J.* (2021) 55:213–9. 10.1080/14017431.2021.1889653 33650449

[247] Pascual-Figal DA Januzzi JL. The biology of ST2: the international ST2 consensus panel. *Am J Cardiol.* (2015) 115:3B–7B. 10.1016/j.amjcard.2015.01.034 25665766

[248] Vílchez JA Pérez-Cuellar M Marín F Gallego P Manzano-Fernández S Valdés M sST2 levels are associated with all-cause mortality in anticoagulated patients with atrial fibrillation. *Eur J Clin Invest.* (2015) 45:899–905. 10.1111/eci.12482 26081996

[249] Ma X Yuan H Luan HX Shi YL Zeng XL Wang Y. Elevated soluble ST2 concentration may involve in the progression of atrial fibrillation. *Clin Chim Acta.* (2018) 480:138–42. 10.1016/j.cca.2018.02.005 29428199

[250] Liu H Wang K Lin YP Liang X Zhao S Li M Role of sST2 in predicting recurrence of atrial fibrillation after radiofrequency catheter ablation. *Pacing Clin Electrophysiol.* (2020) 43:1235–41. 10.1111/pace.14029 32735032

[251] Kim HK Shin DG Lee CH. Effectiveness of soluble st2 study on the progression and therapeutic effects of atrial fibrillation. *J Am Coll Cardiol.* (2020) 75(11 Suppl. 1):326. 10.1016/S0735-1097

[252] Okar S Kaypakli O Sahin DY Koç M. Fibrosis marker soluble ST2 predicts atrial fibrillation recurrence after cryoballoon catheter ablation of nonvalvular paroxysmal atrial fibrillation. *Korean Circ J.* (2018) 48:920–9. 10.4070/kcj.2018.0047 30238709 PMC6158454

[253] Clementy N Piver E Bisson A Andre C Bernard A Pierre B Galectin-3 in atrial fibrillation: mechanisms and therapeutic implications. *Int J Mol Sci.* (2018) 19:976. 10.3390/ijms19040976 29587379 PMC5979515

[254] Sonmez O Ertem FU Vatankulu MA Erdogan E Tasal A Kucukbuzcu S Novel fibro-inflammation markers in assessing left atrial remodeling in non-valvular atrial fibrillation. *Med Sci Monit.* (2014) 20:463–70. 10.12659/MSM.890635 24651058 PMC3965288

[255] Stanojevic D Apostolovic S Stokanovic D Momčilović S Jevtovic-Stoimenov T Salinger-Martinovic S Galectin-3 in acute myocardial infarction patients with atrial fibrillation. *Med Princ Pract.* (2019) 28:284–90. 10.1159/000497611 30726858 PMC6597920

[256] Zhang G Wu Y. Circulating galectin-3 and atrial fibrillation recurrence after catheter ablation: a meta-analysis. *Cardiovasc Ther.* (2019) 2019:4148129. 10.1155/2019/4148129 31772609 PMC6739774

[257] Ramos-Mondragón R Galindo CA Avila G. Role of TGF-beta on cardiac structural and electrical remodeling. *Vasc Health Risk Manag.* (2008) 4:1289–300. 10.2147/vhrm.s3985 19337543 PMC2663446

[258] Verheule S Sat T Everett T IV Engle SK Otten D Rubart-Von Der Lohe M Increased vulnerability to atrial fibrillation in transgenic mice with selective atrial fibrosis caused by overexpression of TGF-β1. *Circ Res.* (2004) 94:1458–65. 10.1161/01.RES.0000129579.59664.9d 15117823 PMC2129102

[259] Letterio JJ Roberts AB. Regulation of immune responses by TGF-β. *Annu Rev Immunol.* (1998) 16:137–61. 10.1146/annurev.immunol.16.1.137 9597127

[260] Zhao S Li M Ju W Gu L Zhang F Chen H Serum level of transforming growth factor beta 1 is associated with left atrial voltage in patients with chronic atrial fibrillation. *Indian Pacing Electrophysiol J.* (2018) 18:95–9. 10.1016/j.ipej.2017.11.001 29155027 PMC5986266

[261] Kim SK Pak HN Park JH Ko KJ Lee JS Choi JI Clinical and serological predictors for the recurrence of atrial fibrillation after electrical cardioversion. *Europace.* (2009) 11:1632–8. 10.1093/europace/eup321 19858160

[262] Ki MR Shin DG Park JS Hong KS Hong IH Park JK Frequency of vacuolating cytotoxin A (VacA)-positive *Helicobacter pylori* seropositivity and TGF-β1 decrease in atrial fibrillation. *Int J Cardiol.* (2010) 145:345–6. 10.1016/j.ijcard.2009.12.009 20034688

[263] Rosenberg MA Maziarz M Tan AY Glazer NL Zieman SJ Kizer JR Circulating fibrosis biomarkers and risk of atrial fibrillation: the cardiovascular health study (CHS). *Am Heart J.* (2014) 167:723–8.e2. 10.1016/j.ahj.2014.01.010 24766983 PMC4060155

[264] Park S Nguyen NB Pezhouman A Ardehali R. Cardiac fibrosis: potential therapeutic targets. *Transl Res.* (2019) 209:121–37. 10.1016/j.trsl.2019.03.001 30930180 PMC6545256

[265] Hudson MP Armstrong PW Ruzyllo W Brum J Cusmano L Krzeski P Effects of selective matrix metalloproteinase inhibitor (PG-116800) to prevent ventricular remodeling after myocardial infarction. Results of the PREMIER (prevention of myocardial infarction early remodeling) trial. *J Am Coll Cardiol.* (2006) 48:15–20. 10.1016/j.jacc.2006.02.055 16814643

[266] Walton KL Johnson KE Harrison CA. Targeting TGF-β mediated MAD signaling for the prevention of fibrosis. *Front Pharmacol.* (2017) 8:461. 10.3389/fphar.2017.00461 28769795 PMC5509761

[267] de Oliveira FL Araújo-Jorge TC de Souza EM de Oliveira GM Degrave WM Feige JJ Oral administration of GW788388, an inhibitor of transforming growth factor beta signaling, prevents heart fibrosis in chagas disease. *PLoS Negl Trop Dis.* (2012) 6:e1696. 10.1371/journal.pntd.0001696 22720109 PMC3373641

[268] Tan SM Zhang Y Connelly KA Gilbert RE Kelly DJ. Targeted inhibition of activin receptor-like kinase 5 signaling attenuates cardiac dysfunction following myocardial infarction. *Am J Physiol Heart Circ Physiol.* (2010) 298:H1415–25. 10.1152/ajpheart.01048.2009 20154262

[269] Engebretsen KVT Skårdal K Bjørnstad S Marstein HS Skrbic B Sjaastad I Attenuated development of cardiac fibrosis in left ventricular pressure overload by SM16, an orally active inhibitor of ALK5. *J Mol Cell Cardiol.* (2014) 76:148–57. 10.1016/j.yjmcc.2014.08.008 25169971

[270] Ikeuchi M Tsutsui H Shiomi T Matsusaka H Matsushima S Wen J Inhibition of TGF-beta signaling exacerbates early cardiac dysfunction but prevents late remodeling after infarction. *Cardiovasc Res.* (2004) 64:526–35. 10.1016/j.cardiores.2004.07.017 15537506

[271] Herbertz S Sawyer JS Stauber AJ Gueorguieva I Driscoll KE Estrem ST Clinical development of galunisertib (LY2157299 monohydrate), a small molecule inhibitor of transforming growth factor-beta signaling pathway. *Drug Design Dev Ther.* (2015) 9:4479–99. 10.2147/DDDT.S86621 26309397 PMC4539082

[272] Gellibert F de Gouville AC Woolven J Mathews N Nguyen VL Bertho-Ruault C Discovery of 4-{4-[3-(pyridin-2-yl)-1H-pyrazol-4-yl]pyridin-2-yl}-N-(tetrahydro-2H-pyran-4-yl)benzamide (GW788388): a potent, selective, and orally active transforming growth factor-beta type I receptor inhibitor. *J Med Chem.* (2006) 49:2210–21. 10.1021/jm0509905 16570917

[273] Petersen M Thorikay M Deckers M van Dinther M Grygielko ET Gellibert F Oral administration of GW788388, an inhibitor of TGF-beta type I and II receptor kinases, decreases renal fibrosis. *Kidney Int.* (2008) 73:705–15. 10.1038/sj.ki.5002717 18075500

[274] King TE Bradford WZ Castro-Bernardini S Fagan EA Glaspole I Glassberg MK A phase 3 trial of pirfenidone in patients with idiopathic pulmonary fibrosis. *N Engl J Med.* (2014) 370:2083–92. 24836312 10.1056/NEJMoa1402582

[275] Edgley AJ Krum H Kelly DJ. Targeting fibrosis for the treatment of heart failure: a role for transforming growth factor-β. *Cardiovasc Ther.* (2012) 30:e30–40. 10.1111/j.1755-5922.2010.00228.x 21883991

[276] Lewis GA Dodd S Clayton D Bedson E Eccleson H Schelbert EB Pirfenidone in heart failure with preserved ejection fraction: a randomized phase 2 trial. *Nat Med.* (2021) 27:1477–82. 10.1038/s41591-021-01452-0 34385704

[277] Platten M Ho PP Youssef S Fontoura P Garren H Hur EM Treatmentof autoimmune neuroinflammation with a synthetic tryptophan metabolite. *Science.* (2005) 310:850–5. 10.1126/science.1117634 16272121

[278] Kogure F Ishizaki M Saiga T. Long-term clinical study of tranilast ophthalmicsolution on vernal conjunctivitis. *J Clin Ther Med.* (1993) 9:429–41.

[279] Shioda H. A double-blind controlled trial of N-(3,4-dimethoxycinnamoyl) anthranilic acid on children with bronchial asthma, N-5 study group in chil-dren. *Allergy.* (1979) 34:213–9. 10.1111/j.1398-9995.1979.tb01701.x 391094

[280] Horiuchi Y Bae S Katayama I. Uncontrollable prurigo nodularis effectively treated by roxithromycin and tranilast. *J Drugs Dermatol.* (2006) 5:363–5. 16673805

[281] Isaji M Nakajoh J. Naito selective inhibition of collagen accumulation by N-(3,4-dimethoxycinnamoy1) anthranilic acid (N-5’) in granulation tissue. *Biochem Pharmacol.* (1987) 36:469–74. 10.1016/0006-2952(87)90353-4 2435288

[282] Suzawa H Kikuchi S Arai N Koda A. The mechanism involved in the inhibitory action of tranilast on collagen biosynthesis of keloid fibroblasts. *Jpn J Pharmacol.* (1992) 60:91–6. 10.1254/jjp.60.91 1282576

[283] Taniguchi S Yorifuji T Hamada T. Treatment of linear localized scleroderma with the anti-allergic drug, tranilast. *Clin Exp Dermatol.* (1994) 19:391–3. 10.1111/j.1365-2230.1994.tb02689.x 7525127

[284] Yamada H Tajima S Nishikawa T Murad S Pinnell SR. Tranilast a selec-tive inhibitor of collagen synthesis in human skin fibroblasts. *J Biochem.* (1994) 116:892–7. 10.1093/oxfordjournals.jbchem.a124612 7533764

[285] Yashiro M Chung YS Sowa M. Tranilast (N-(3,4-dimethoxycinnamoyl)anthranilic acid) down-regulates the growth inhibition between gastric cancer cells and fibroblasts. *Anticancer Res.* (1997) 17:895–900. 9137424

[286] Isaji M Kikuchi S Miyata H Ajisawa Y Araki-Inazawa K Tsukamoto Y Inhibitory effects of tranilast on the proliferation and functionof human pterygium-derived fibroblasts. *Cornea.* (2000) 19:364–8. 10.1097/00003226-200005000-00021 10832700

[287] Fang L Murphy AJ Dart AM. A clinical perspective of anti-fibrotic therapies for cardiovascular disease. *Front Pharmacol.* (2017) 8:186. 10.3389/fphar.2017.00186 28428753 PMC5382201

[288] Yu L Ruifrok WP Meissner M Bos EM van Goor H Sanjabi B Genetic and pharmacological inhibition of galectin-3 prevents cardiac remodeling by interfering with myocardial fibrogenesis. *Circ Heart Fail.* (2013) 6:107–17. 10.1161/CIRCHEARTFAILURE.112.971168 23230309

[289] de Boer RA Voors AA Muntendam P van Gilst WH van Veldhuisen DJ. Galectin-3: a novel mediator of heart failure development and progression. *Eur J Heart Fail.* (2009) 11:811–7. 10.1093/eurjhf/hfp097 19648160

[290] Prasad SK Dargie HJ Smith GC Barlow MM Grothues F Groenning BA Comparison of the dual receptor endothelin antagonist enrasentan with enalapril in asymptomatic left ventricular systolic dysfunction: a cardiovascular magnetic resonance study. *Heart.* (2006) 92:798–803. 10.1136/hrt.2004.049734 16339819 PMC1860639

[291] Anand I McMurray J Cohn JN Konstam MA Notter T Quitzau K Long-term effects of darusentan on left-ventricular remodelling and clinical outcomes in the EndothelinA receptor antagonist trial in heart failure (EARTH): randomised, double-blind, placebo-controlled trial. *Lancet.* (2004) 364:347–54. 10.1016/S0140-6736(04)16723-8 15276394

[292] McDonagh T Metra M Adamo M Gardner RS Baumbach A Böhm M 2021 ESC Guidelines for the diagnosis and treatment of acute and chronic heart failure: developed by the task force for the diagnosis and treatment of acute and chronic heart failure of the European society of cardiology (ESC) with the special contribution of the heart failure association (HFA) of the ESC. *Eur Heart J.* (2021) 42:3599–726. 10.1093/eurheartj/ehab368 34447992

[293] Milliez P Deangelis N Rucker-Martin C Leenhardt A Vicaut E Robidel E Spironolactone reduces fibrosis of dilated atria during heart failure in rats with myocardial infarction. *Eur Heart J.* (2005) 26:2193–9. 10.1093/eurheartj/ehi478 16141258

[294] Lee KW Everett TH IV Rahmutula D Guerra JM Wilson E Ding C Pirfenidone prevents the development of a vulnerable substrate for atrial fibrillation in a canine model of heart failure. *Circulation.* (2006) 114:1703–12. 10.1161/CIRCULATIONAHA.106.624320 17030685 PMC2129103

[295] Richter B Derntl M Marx M Lercher P Gössinger HD. Therapy with angiotensin-converting enzyme inhibitors, angiotensin II receptor blockers, and statins: no effect on ablation outcome after ablation of atrial fibrillation. *Am Heart J.* (2007) 153:113–9. 10.1016/j.ahj.2006.09.006 17174648

[296] Leonardi M Bissett J. Prevention of atrial fibrillation. *Curr Opin Cardiol.* (2005) 20:417–23. 16093761 10.1097/01.hco.0000172703.44898.27

[297] Savelieva I Kakouros N Kourliouros A Camm AJ. Upstream therapies for management of atrial fibrillation: review of clinical evidence and implications for European society of cardiology guidelines. Part II: secondary prevention. *Europace.* (2011) 13:610–25. 10.1093/europace/eur023 21515595

[298] Al Chekakie MO Akar JG Wang F Al Muradi H Wu J Santucci P The effects of statins and renin–angiotensin system blockers on atrial fibrillation recurrence following antral pulmonary vein isolation. *J Cardiovasc Electrophysiol.* (2007) 18:942–6. 10.1111/j.1540-8167.2007.00887.x 17593228

[299] Macfarlane PW Murray H Sattar N Stott DJ Ford I Buckley B The incidence and risk factors for new onset atrial fibrillation in the PROSPER study. *Europace.* (2011) 13:634–9. 10.1093/europace/eur016 21325345

[300] Fauchier L Clementy N Babuty D. Statin therapy and atrial fibrillation: systematic review and updated meta-analysis of published randomized controlled trials. *Curr Opin Cardiol.* (2013) 28:7–18. 10.1097/HCO.0b013e32835b0956 23160338

[301] Yang Q Qi X Li Y. The preventive effect of atorvastatin on atrial fibrillation: a meta-analysis of randomized controlled trials. *BMC Cardiovasc Disord.* (2014) 14:99. 10.1186/1471-2261-14-99 25117689 PMC4135360

[302] Shah A Gandhi D Srivastava S Shah KJ Mansukhani R. Heart failure: a class review of pharmacotherapy. *P T.* (2017) 42:464–72. 28674474 PMC5481297

[303] Mann DL. Inflammatory mediators and the failing heart: past, present, and the foreseeable future. *Circ Res.* (2002) 91:988–98. 10.1161/01.res.0000043825.01705.1b 12456484

[304] Engelhardt S Hein L Wiesmann F Lohse MJ. Progressive hypertrophy and heart failure in beta1-adrenergic receptor transgenic mice. *Proc Natl Acad Sci USA.* (1999) 96:7059–64. 10.1073/pnas.96.12.7059 10359838 PMC22055

[305] Turner NA Porter KE Smith WH White HL Ball SG Balmforth AJ. Chronic beta2-adrenergic receptor stimulation increases proliferation of human cardiac fibroblasts via an autocrine mechanism. *Cardiovasc Res.* (2003) 57:784–92. 10.1016/s0008-6363(02)00729-0 12618240

[306] Le DE Pascotto M Leong-Poi H Sari I Micari A Kaul S. Anti-inflammatory and pro-angiogenic effects of beta blockers in a canine model of chronic ischemic cardiomyopathy: comparison between carvedilol and metoprolol. *Basic Res Cardiol.* (2013) 108:384. 10.1007/s00395-013-0384-7 24072434 PMC3867789

[307] Lacolley P Labat C Pujol A Delcayre C Benetos A Safar M. Increased carotid wall elastic modulus and fibronectin in aldosterone-salt-treated rats: effects of eplerenone. *Circulation.* (2002) 106:2848–53. 10.1161/01.cir.0000039328.33137.6c 12451013

[308] Blasi ER Rocha R Rudolph AE Blomme EA Polly ML McMahon EG. Aldosterone/salt induces renal inflammation and fibrosis in hypertensive rats. *Kidney Int.* (2003) 63:1791–800. 10.1046/j.1523-1755.2003.00929.x 12675855

[309] Brilla CG Matsubara LS Weber KT. Anti-aldosterone treatment and the prevention of myocardial fibrosis in primary and secondary hyperaldosteronism. *J Mol Cell Cardiol.* (1993) 25:563–75. 10.1006/jmcc.1993.1066 8377216

[310] Brown NJ. Contribution of aldosterone to cardiovascular and renal inflammation and fibrosis. *Nat Rev Nephrol.* (2013) 9:459–69. 10.1038/nrneph.2013.110 23774812 PMC3922409

[311] Kupfahl C Pink D Friedrich K Zurbrügg HR Neuss M Warnecke C Angiotensin II directly increases transforming growth factor beta1 and osteopontin and indirectly affects collagen mRNA expression in the human heart. *Cardiovasc Res.* (2000) 46:463–75. 10.1016/s0008-6363(00)00037-7 10912457

[312] Ambari AM Setianto B Santoso A Radi B Dwiputra B Susilowati E Angiotensin converting enzyme inhibitors (ACEIs) decrease the progression of cardiac fibrosis in rheumatic heart disease through the inhibition of IL-33/sST2. *Front Cardiovasc Med.* (2020) 7:115. 10.3389/fcvm.2020.00115 32850979 PMC7399157

[313] Wolf G Ziyadeh FN Stahl RA. Angiotensin II stimulates expression of transforming growth factor beta receptor type II in cultured mouse proximal tubular cells. *J Mol Med (Berl).* (1999) 77:556–64. 10.1007/s001099900028 10494801

[314] De Albuquerque DA Saxena V Adams DE Boivin GP Brunner HI Witte DP An ACE inhibitor reduces Th2 cytokines and TGF-beta1 and TGF-beta2 isoforms in murine lupus nephritis. *Kidney Int.* (2004) 65:846–59. 10.1111/j.1523-1755.2004.00462.x 14871404 PMC2291513

[315] Perez O Garvin A Hale T. Transient ACE-inhibitor treatment produces persistent change in cardiac fibroblast physiology. *FASEB J.* (2018) 32:867.4. 10.1096/fasebj.2018.32.1_supplement.867.4

[316] Maskito VJ Anniwati L. The difference between st2 and nt-pro bnp concentrations before and after-treatment of ace-inhibitors in nyha iii-iv heart failure patients. *Indones J Clin Pathol Med Lab.* (2019) 26:11–7. 10.24293/ijcpml.v26i1.1366

[317] Oesterle A Laufs U Liao JK. Pleiotropic effects of statins on the cardiovascular system. *Circ Res.* (2017) 120:229–43. 10.1161/CIRCRESAHA.116.308537 28057795 PMC5467317

[318] Vasarmidi E Tsitoura E Spandidos DA Tzanakis N Antoniou KM. Pulmonary fibrosis in the aftermath of the Covid-19 era (review). *Exp Ther Med.* (2020) 20:2557–60. 10.3892/etm.2020.8980 32765748 PMC7401793

[319] Aydın K Gülçelik NE Tuncel M Balcı C Akın S Çınar N Thyroid volumes and serum VEGF levels in dyslipidemic patients: effects of statin treatment. *Turk J Med Sci.* (2019) 49:738–45. 10.3906/sag-1708-106 31203592 PMC7018346

[320] Yildirim M Kayalar O Atahan E Oztay F. Anti-fibrotic effect of atorvastatin on the lung fibroblasts and myofibroblasts. *Eur Respir J.* (2018) 52:A991. 10.1183/13993003.congress-2018.PA991

[321] Saewong S Thammasitboon K Wattanaroonwong N. Simvastatin induces apoptosis and disruption of the actin cytoskeleton in human dental pulp cells and periodontal ligament fibroblasts. *Arch Oral Biol.* (2013) 58:964–74. 10.1016/j.archoralbio.2013.03.002 23561831

[322] Saito A Horie M Nagase T. TGF-β signaling in lung health and disease. *Int J Mol Sci.* (2018) 19:2460. 10.3390/ijms19082460 PMC6121238 30127261

[323] Yang T Chen M Sun T. Simvastatin attenuates TGF-β1-induced epithelial-mesenchymal transition in human alveolar. Epithelial cells. *Cell Physiol Biochem.* (2013) 31:863–74. 10.1159/000350104 23817018

[324] Kim SK Park JH Kim JY Choi JI Joung B Lee M-H High plasma concentrations of transforming growth factor-β and tissue inhibitor of metalloproteinase-1. *Circ J.* (2011) 75:557–64. 10.1253/circj.CJ-10-0758 21186331

[325] Wei YH Liao SL Wang SH Wang CC Yang CH. Simvastatin and ROCK inhibitor Y-27632 inhibit myofibroblast differentiation of graves’ ophthalmopathy-derived orbital fibroblasts via RhoA-mediated ERK and p38 signaling pathways. *Front Endocrinol (Lausanne).* (2021) 11:607968. 10.3389/fendo.2020.607968 33597925 PMC7883643

[326] Essig M Nguyen G Prie D Escoubet B Sraer JD Friedlander G. 3-Hydroxy-3-methylglutaryl coenzyme A reductase inhibitors increase fibrinolytic activity in rat aortic endothelial cells. Role of geranylgeranylation and Rho proteins. *Circ Res.* (1998) 83:683–90. 10.1161/01.res.83.7.683 9758637

[327] Martino A Pezzi L Magnano R Salustri E Penco M Calo L. Omega 3 and atrial fibrillation: where are we? *World J Cardiol.* (2016) 8:114–9. 10.4330/wjc.v8.i2.114 26981208 PMC4766263

[328] Mohammed K Kowey PR Musco S. Adjuvant therapy for atrial fibrillation. *Future Cardiol.* (2010) 6:67–81. 20014988 10.2217/fca.09.57

[329] Andrade JG Wells GA Deyell MW Bennett M Essebag V Champagne J Cryoablation or drug therapy for initial treatment of atrial fibrillation. *N Engl J Med.* (2021) 384:305–15. 10.1056/nejmoa2029980 33197159

[330] Tilz RR Rillig A Thum AM Arya A Wohlmuth P Metzner A Catheter ablation of long-standing persistent atrial fibrillation: 5-year outcomes of the Hamburg sequential ablation strategy. *J Am Coll Cardiol.* (2012) 60:1921–9. 10.1016/j.jacc.2012.04.060 23062545

[331] Scherr D Khairy P Miyazaki S Aurillac-Lavignolle V Pascale P Wilton SB Five-year outcome of catheter ablation of persistent atrial fibrillation using termination of atrial fibrillation as a procedural endpoint. *Circ Arrhythm Electrophysiol.* (2015) 8:18–24. 10.1161/CIRCEP.114.001943 25528745

[332] de Vos CB Pisters R Nieuwlaat R Prins MH Tieleman RG Coelen RJS Progression from paroxysmal to persistent atrial fibrillation. clinical correlates and prognosis. *J Am Coll Cardiol.* (2010) 55:725–31. 10.1016/j.jacc.2009.11.040 20170808

[333] De Sisti A Leclercq JF Halimi F Fiorello P Bertrand C Attuel P. Evaluation of time course and predicting factors of progression of paroxysmal or persistent atrial fibrillation to permanent atrial fibrillation. *Pacing Clin Electrophysiol.* (2014) 37:345–55. 10.1111/pace.12264 24236932

[334] Padfield GJ Steinberg C Swampillai J Qian H Connolly SJ Dorian P Progression of paroxysmal to persistent atrial fibrillation: 10-year follow-up in the Canadian registry of atrial fibrillation. *Heart Rhythm.* (2017) 14:801–7. 10.1016/j.hrthm.2017.01.038 28232263

[335] Knight BP Novak PG Sangrigoli R Champagne J Dubuc M Adler SW Long-term outcomes after ablation for paroxysmal atrial fibrillation using the second-generation cryoballoon: final results from STOP AF post-approval study. *JACC Clin Electrophysiol.* (2019) 5:306–14. 10.1016/j.jacep.2018.11.006 30898232

[336] Hermida JS Chen J Meyer C Iacopino S Arena G Pavlovic N Cryoballoon catheter ablation versus antiarrhythmic drugs as a first-line therapy for patients with paroxysmal atrial fibrillation: rationale and design of the international Cryo-FIRST study. *Am Heart J.* (2020) 222:64–72. 10.1016/j.ahj.2019.12.006 32018203

[337] Kirchhof P Camm AJ Goette A Brandes A Eckardt L Elvan A Early rhythm-control therapy in patients with atrial fibrillation. *N Engl J Med.* (2020) 383:1305–16. 10.1056/nejmoa2019422 32865375

[338] Kuck KH Lebedev DS Mikhaylov EN Romanov A Gellér L Kalējs O Catheter ablation or medical therapy to delay progression of atrial fibrillation: the randomized controlled atrial fibrillation progression trial (ATTEST). *Europace.* (2021) 23:362–9. 10.1093/europace/euaa298 33330909 PMC7947582

[339] Teh AW Kistler PM Lee G Medi C Heck PM Spence SJ Electroanatomic remodeling of the left atrium in paroxysmal and persistent atrial fibrillation patients without structural heart disease. *J Cardiovasc Electrophysiol.* (2012) 23:232–8. 10.1111/j.1540-8167.2011.02178.x 21955090

[340] Cochet H Dubois R Yamashita S Al Jefairi N Berte B Sellal JM Relationship between fibrosis detected on late gadolinium-enhanced cardiac magnetic resonance and re-entrant activity assessed with electrocardiographic imaging in human persistent atrial fibrillation. *JACC Clin Electrophysiol.* (2018) 4:17–29. 10.1016/j.jacep.2017.07.019 29479568 PMC5824731

[341] Jalife J Berenfeld O Mansour M. Mother rotors and fibrillatory conduction: a mechanism of atrial fibrillation. *Cardiovasc Res.* (2002) 54:204–16. 10.1016/s0008-6363(02)00223-7 12062327

[342] Haissaguerre M Hocini M Denis A Shah AJ Komatsu Y Yamashita S Driver domains in persistent atrial fibrillation. *Circulation.* (2014) 130:530–8. 10.1161/CIRCULATIONAHA.113.005421) 25028391

[343] Jadidi AS Cochet H Shah AJ Kim SJ Duncan E Miyazaki S Inverse relationship between fractionated electrograms and atrial fibrosis in persistent atrial fibrillation: combined magnetic resonance imaging and high-density mapping. *J Am Coll Cardiol.* (2013) 62:802–12. 10.1016/j.jacc.2013.03.081 23727084

[344] Rhee TM Lee SR Cha MJ Choi EK Oh S. Association of complex fractionated electrograms with atrial myocardial thickness and fibrosis. *Int J Arrhythm.* (2018) 19:6–13. 10.1371/journal.pone.0166972 27875591 PMC5119821

[345] Narayan SM Krummen DE Rappel WJ. Clinical mapping approach to diagnose electrical rotors and focal impulse sources for human atrial fibrillation. *J Cardiovasc Electrophysiol.* (2012) 23:447–54. 10.1111/j.1540-8167.2012.02332.x 22537106 PMC3418865

[346] Narayan SM Krummen DE Shivkumar K Clopton P Rappel W-J Miller JM. Treatment of atrial fibrillation by the ablation of localized sources: confirm conventional ablation for atrial fibrillation with or without focal and rotor modulation) trial. *J Am Coll Cardiol.* (2012) 60:628–36. 22818076 10.1016/j.jacc.2012.05.022 PMC3416917

[347] Mohanty S Mohanty P Trivedi C Gianni C Della Rocca DG Di Biase L Long-term outcome of pulmonary vein isolation with and without focal impulse and rotor modulation mapping: insights from a meta-analysis. *Circ Arrhythm Electrophysiol.* (2018) 11:e005789. 10.1161/CIRCEP.117.005789 29545360

[348] Kottkamp H Berg J Bender R Rieger A Schreiber D. Box isolation of fibrotic areas (BIFA): a patient-tailored substrate modification approach for ablation of atrial fibrillation. *J Cardiovasc Electrophysiol*. (2016) 27:22–30. 10.1111/jce.12870 26511713

[349] Yang G Yang B Wei Y Zhang F Ju W Chen H Catheter ablation of nonparoxysmal atrial fibrillation using electrophysiologically guided substrate modification during sinus rhythm after pulmonary vein isolation. *Circ Arrhythm Electrophysiol*. (2016) 9:e003382. 10.1161/CIRCEP.115.003382 26857907

[350] Yamaguchi T Tsuchiya T Fukui A Kawano Y Otsubo T Takahashi Y Impact of the extent of low-voltage zone on outcomes after voltage-based catheter ablation for persistent atrial fibrillation. *J Cardiol*. (2018) 72:427–33. 10.1016/j.jjcc.2018.04.010 29807864

[351] Jadidi AS Lehrmann H Keyl C Sorrel J Markstein V Minners J Ablation of persistent atrial fibrillation targeting low-voltage areas with selective activation characteristics. *Circ Arrhythm Electrophysiol*. (2016) 9:e002962. 10.1161/circep.115.002962 26966286

[352] Rolf S Kircher S Arya A Eitel C Sommer P Richter S Tailored atrial substrate modification based on low-voltage areas in catheter ablation of atrial fibrillation. *Circ Arrhythm Electrophysiol*. (2014) 7:825–33. 10.1161/CIRCEP.113.001251 25151631

[353] Scott PA Silberbauer J Murgatroyd FD. The impact of adjunctive complex fractionated atrial electrogram ablation and linear lesions on outcomes in persistent atrial fibrillation: a meta-analysis. *Europace.* (2016) 18:359–67. 10.1093/europace/euv351 26559915

[354] Blandino A Bianchi F Grossi S Biondi-Zoccai G Conte MR Gaido L Left atrial substrate modification targeting low-voltage areas for catheter ablation of atrial fibrillation: a systematic review and meta-analysis. *Pacing Clin Electrophysiol.* (2017) 40:199–212. 10.1111/pace.13015 28054377

[355] Ahmed-Jushuf F Murgatroyd F Dhillon P Scott PA. The impact of the presence of left atrial low voltage areas on outcomes from pulmonary vein isolation. *J Arrhythm.* (2019) 35:205–14. 10.1002/joa3.12174 31007784 PMC6457382

[356] Masuda M Asai M Iida O Okamoto S Ishihara T Nanto K Additional low-voltage-area ablation in patients with paroxysmal atrial fibrillation: results of the randomized controlled VOLCANO trial. *J Am Heart Assoc.* (2020) 9:e015927. 10.1161/JAHA.120.015927 32578466 PMC7670527

[357] Marrouche NF Wazni O McGann C Greene T Dean JM Dagher L Effect of MRI-Guided fibrosis ablation vs conventional catheter ablation on atrial arrhythmia recurrence in patients with persistent atrial fibrillation: the DECAAF II Randomized Clinical Trial. *JAMA* (2022) 327:2296–2305. 10.1001/jama.2022.8831 35727277 PMC9214588

[358] Haissaguerre M Shah AJ Cochet H Hocini M Dubois R Efimov I Intermittent drivers anchoring to structural heterogeneities as a major pathophysiological mechanism of human persistent atrial fibrillation. *J Physiol.* (2016) 594:2387–98. 10.1113/JP270617 26890861 PMC4850206

